# The importance of utilizing travel history metadata for informative phylogeographical inferences: a case study of early SARS-CoV-2 introductions into Australia

**DOI:** 10.1099/mgen.0.001099

**Published:** 2023-08-31

**Authors:** Ashleigh F. Porter, Leo Featherstone, Courtney R. Lane, Norelle L. Sherry, Monica L. Nolan, David Lister, Torsten Seemann, Sebastian Duchene, Benjamin P. Howden

**Affiliations:** ^1^​ Department of Microbiology and Immunology, The University of Melbourne at The Peter Doherty Institute for Infection and Immunity, Melbourne, VIC, Australia; ^2^​ Microbiological Diagnostic Unit Public Health Laboratory, The University of Melbourne at The Peter Doherty Institute for Infection and Immunity, Melbourne, VIC, Australia; ^3^​ Department of Infectious Diseases, Austin Health, Heidelberg, VIC, Australia; ^4^​ Victorian Department of Health, VIC, Australia; ^5^​ Centre for Pathogen Genomics, The University of Melbourne, Melbourne, VIC, Australia

**Keywords:** Bayesian phylogeography, COVID-19, genomics, metadata, public health, SARS-CoV-2

## Abstract

Inferring the spatiotemporal spread of severe acute respiratory syndrome coronavirus 2 (SARS-CoV-2) via Bayesian phylogeography has been complicated by the overwhelming sampling bias present in the global genomic dataset. Previous work has demonstrated the utility of metadata in addressing this bias. Specifically, the inclusion of recent travel history of SARS-CoV-2-positive individuals into extended phylogeographical models has demonstrated increased accuracy of estimates, along with proposing alternative hypotheses that were not apparent using only genomic and geographical data. However, as the availability of comprehensive epidemiological metadata is limited, many of the current estimates rely on sequence data and basic metadata (i.e. sample date and location). As the bias within the SARS-CoV-2 sequence dataset is extensive, the degree to which we can rely on results drawn from standard phylogeographical models (i.e. discrete trait analysis) that lack integrated metadata is of great concern. This is particularly important when estimates influence and inform public health policy. We compared results generated from the same dataset, using two discrete phylogeographical models: one including travel history metadata and one without. We utilized sequences from Victoria, Australia, in this case study for two unique properties. Firstly, the high proportion of cases sequenced throughout 2020 within Victoria and the rest of Australia. Secondly, individual travel history was collected from returning travellers in Victoria during the first wave (January to May) of the coronavirus disease 2019 (COVID-19) pandemic. We found that the implementation of individual travel history was essential for the estimation of SARS-CoV-2 movement via discrete phylogeography models. Without the additional information provided by the travel history metadata, the discrete trait analysis could not be fit to the data due to numerical instability. We also suggest that during the first wave of the COVID-19 pandemic in Australia, the primary driving force behind the spread of SARS-CoV-2 was viral importation from international locations. This case study demonstrates the necessity of robust genomic datasets supplemented with epidemiological metadata for generating accurate estimates from phylogeographical models in datasets that have significant sampling bias. For future work, we recommend the collection of metadata in conjunction with genomic data. Furthermore, we highlight the risk of applying phylogeographical models to biased datasets without incorporating appropriate metadata, especially when estimates influence public health policy decision making.

## Data Summary

The genomic data used in this study were collected from the GISAID repository. Accession numbers, along with the corresponding originating and submitting laboratories, have been provided in the Supplementary Material. The bioinformatics workflow is available on github.com/aporter704/Phylogeo.

**Impact Statement**

Infectious disease sequence datasets that have substantial sampling bias are incompatible with standard phylogeographical methods. The global severe acute respiratory syndrome coronavirus 2 (SARS-CoV-2) genomic dataset is predominantly made up of sequences from high-income countries. As the data gathered from phylogeographical models have proven essential for informing public health measures, there is a need for caution in generating these estimates. Previously, innovative methodology has implemented other forms of data in phylogeography models to reduce the impact of sampling bias. This study highlights the importance of collecting and utilizing metadata, specifically individual travel history (the collection of a SARS-CoV-2-positive individual’s recent travel locations), in phylogeographical modelling of SARS-CoV-2. We demonstrate that the inclusion of travel history metadata is essential for generating accurate estimates of SARS-CoV-2 movement during the first-wave of coronavirus disease 2019 (COVID-19) in Australia. This study exemplifies the importance of collecting and sharing metadata for phylogeographical modelling to inform ongoing and future pandemic responses.

## Introduction

Understanding the spatiotemporal spread of severe acute respiratory syndrome coronavirus 2 (SARS-CoV-2) during the ongoing coronavirus disease 2019 (COVID-19) pandemic has been complicated by incomplete and biased sampling, particularly during the early months of 2020, the first wave [1–4]. The first wave of SARS-CoV-2 in Australia was characterized by a polyclonal surge of COVID-19 cases associated with international travel, with a relatively low rate of SARS-CoV-2 infections in the community, attributed to public health interventions. The situation is unique for several reasons: firstly, being an island nation, all virus incursions occurred via international travel and were controlled by rapidly enforced border closures, along with testing and isolation of infected travellers. Additionally, Australia had a high case ascertainment rate and sequenced a substantial proportion of detected COVID-19 cases (up to 80 % in the state of Victoria) [5]. This was partly due to low case numbers during the first year of the pandemic, where infection and mortality rates in Australia were substantially lower than in other comparable countries within the Organisation for Economic Co-operation and Development (OECD) [6].

**Fig. 1.**
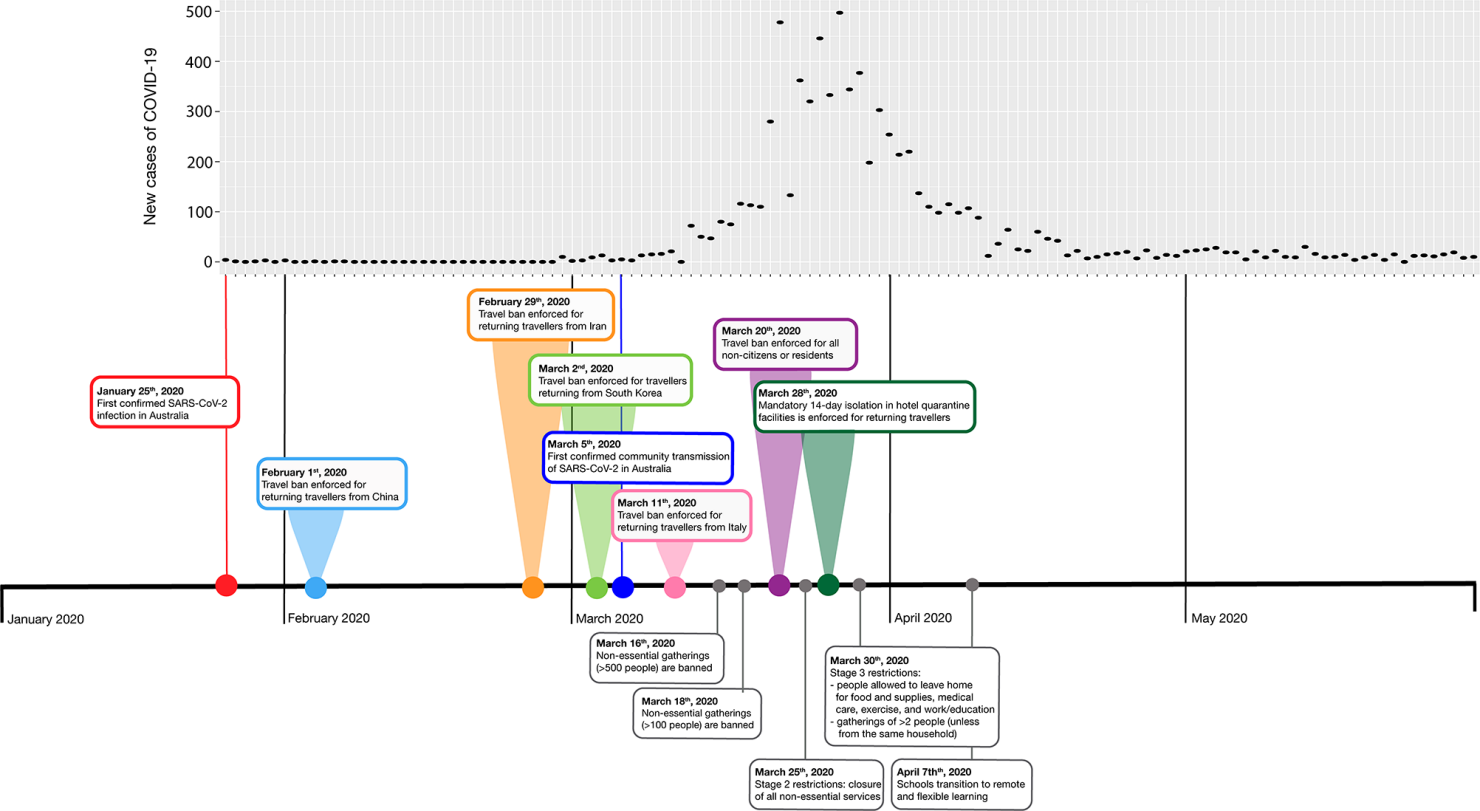
An overview of restrictions implemented by Australian and Victorian governments during the first-wave of SARS-CoV-2. From January to May 2020, the Australian government enforced several border and quarantine restrictions to attempt to halt the spread of SARS-CoV-2 into Australia (coloured bubbles, top). The Victorian government introduced a range of social and movement restrictions to attempt to slow the spread of SARS-CoV-2 within the state (grey bubbles, below).

During the first wave, several non-pharmaceutical interventions (NPIs) were implemented in Australia to slow the spread of SARS-CoV-2. Within the state of Victoria, a state of emergency was declared on 16 March 2020, which enabled mandatory case isolation, quarantine of close contacts, restrictions on non-essential services, social distancing requirements, and the scaling of substantial resources to facilitate compliance and enforcement activities (Fig. 1). As an additional precaution, travel bans were enforced for travellers returning from countries with early high-case rates, including mainland PR China, Iran, the Republic of Korea and Italy. The border was restricted to all non-residents or citizens, and 14 day hotel quarantine orders were enforced in late March 2020 (Fig. 1).

The conditions of the first wave of SARS-CoV-2 in Australia present an interesting case when considering the movement (i.e. importations and exportations) of the virus around the globe. In the context of Bayesian phylogeography, we refer to the movement of individuals or lineages between different geographical locations (or ‘demes’) as importation and exportation events. Based on the low prevalence of SARS-CoV-2 circulating within the Australian community, it is anticipated that this wave was driven by importations of the virus from global locations. The first wave is further distinguished from later waves of the pandemic by its high diversity of lineages and relatively low proportion of community transmission, which suggests there were minimal exportations of the virus from Australia to other locations. Lastly, within the state of Victoria, both recent travel history and sequence data were available for approximately one-third of the total dataset during the first wave.

The spread and evolution of infectious agents, such as SARS-CoV-2, are usually examined through complex models that incorporate sequence data and epidemiological metadata, such as sampling location. The combination of epidemiological and sequence data to inform models, known as phylodynamics, has been used to infer introductions of SARS-CoV-2 lineages, along with community transmission and cryptic transmission (undetected or asymptomatic cases circulating in a community) [5, 7]. The pandemic response has been heavily influenced by the estimates generated from phylodynamics, such as reproductive number and growth rates of SARS-CoV-2 lineages [5].

The inclusion of epidemiological data has proven remarkably useful in improving estimates from both phylodynamic and phylogeographical models [8–10]. For example, the combined epidemiological metadata and sequence data were used during the Lassa virus outbreak in West Africa to estimate the direction and temporal spread of the virus [11]. While phylodynamics became a major element of the toolset used to model the COVID-19 pandemic [2, 4, 12], there are two major issues with the SARS-CoV-2 dataset. Firstly, despite the global effort in generating SARS-CoV-2 sequences, it has been limited by the lack of sequence diversity due to fact that transmission occurs over a shorter timescale than that over which substitutions accumulate. Additionally, the global dataset has an overwhelming spatiotemporal sampling bias [10]. For example, there were almost 1 million cases in South America during the first 5 months of pandemic, yet fewer than 3000 samples were sequenced (0.3 % of cases sampled). In comparison, due to its low case load, Australia had an equivalent sequenced proportion of samples of 54 % at the same time point (Table 1). The high sequencing proportion observed in Australia has the potential to distort estimates of the spatiotemporal movement of SARS-CoV-2, with Australia appearing as an epicentre of the pandemic, and a major exporter of the virus to other locations. Secondly, the epidemiological metadata required to supplement the SARS-CoV-2 genomic dataset are scarce, poor quality and often inaccessible [4].

**Table 1.** An overview of the number of sequences collected, number of cases and estimated sampling proportion in each deme (geographical location), along with the number of sequences from each deme used in our analysis

Deme	No. of sequences^∗^	No. of cases†	Estimated sampling proportion (%)	No. of sequences collected from global GISAID dataset	No. of Victorian sequences with travel history listed
UK	4369	256 701	1.7	80	20
Europe‡	40 728	1 700 684	2.4	40	13
PR China	802	86 367	0.9	6	2
Asia§	9547	1 055 600	0.9	16	9
South America	2639	902 985	0.3	2	0
North America	30 193	2 021 665	1.5	95	39
Africa	1343	404 891	0.3	2	0
Australia	3889	7202	54	13	0
New Zealand	732	1504	49	17	17
Total global sequences				193	
Total Victorian sequences (with travel history)				100	
**Total sequences used in analysis**				**293**	

^∗^Available on GISAID (earlier than 1 June 2020).

†Cumulative cases 31 May 2020 (ourworldindata.org/coronavirus) [31].

‡Excluding UK.

§Excluding PR China.

**Table 2.** An overview of the number of Australian sequences used in our analysis, including sequences without travel history, and those with travel history (and the deme listed for travel history)

Australian sequences without travel history	13	
**Australian sequences with travel history**	**100**	
	*Travel history deme*	*Number of sequences*
	Europe	13
	Asia	9
	UK	20
	China	2
	Africa	0
	North America	39
	South America	0
	New Zealand	17

Recent innovative methods have been developed to adapt phylodynamic and phylogeographical models to manage the sampling bias present in the SARS-CoV-2 genomic dataset. One major improvement in methodology is the implementation of individual travel history metadata into phylogeographical inference, facilitating more realistic hypotheses of the directionality of the spread of SARS-CoV-2 [10]. This work provided alternative hypotheses for routes of virus transmission that were likely from an epidemiological perspective but could not be drawn from confirmed-case genomic datasets alone. Importantly, estimates generated from these analyses can provide additional insights into the effectiveness of border closures. These methods could be implemented into outbreak response planning for future epidemics and pandemics.

The Victorian dataset was nominated as it provides an ideal case study for exploring the spatiotemporal spread of SARS-CoV-2 in the first wave of the COVID-19 pandemic, along with assessing the benefit of epidemiological metadata in phylogeographical models. We implemented two forms of discrete trait analysis (DTA) phylogeographical inference to explore the spread of SARS-CoV-2 into Victoria during the first wave: one applying novel methodology [10] to allow the inclusion of individual travel history (DTA-TH) and one without travel history (DTA).

We explored the global exportation and importation events of SARS-CoV-2 during the early months of the pandemic (until 1 June 2020), focusing on inferring the putative number of importations into Australia, and their respective sources.

## Methods

### Data curation

As a natural progression from previous work [5, 13], we explored the Australian importation and exportation of sequences by utilizing a global genomic dataset supplemented with sequences that had associated individual travel history metadata collected in Victoria, Australia. As our aim was to focus on the first wave of SARS-CoV-2 in Australia, our dataset was restricted to sequences collected before the 1 June 2020. Of the total number of sequences collected in Victoria in that period (*n*=1,077), a subset reported individual travel history (*n*=625). Of that subset, we only used the sequences that listed a country (or countries) within a singular deme, excluding travel history from international oceans (i.e. cruise ships) (*n*=428). We chose to exclude international oceans from this analysis, as we assumed that those samples might have different dynamics (e.g. cruise ships with longer-term transmission on board) than other sources of the virus. We restricted the dataset with travel history metadata to only include those with complete, high-coverage genomes (*n*=257), and randomly selected 100 sequences to use in our analysis to reduce the impact of oversampling sequences collected in Australia. For these 100 sequences, we split the travel history location into 9 geographical groups, or demes (see Tables 1 and 2)).

For global sequence data, we randomly sampled 200 complete and high-sequencing coverage SARS-CoV-2 genomes from GISAID (before 1 June 2020) [14, 15] using GISAIDR [16]. Importantly, this dataset includes Australian sequences that do not report travel history (Tables 1 and 2). The sampling locations recorded for these genomes were designated into the demes described in Table 1. The accession numbers and metadata for GISAID sequences used in this study are available in Table S1 and Table S3 (available in the online version of this article).

The final sequence dataset, excluding outliers (*n*=293, Table 1), was aligned in MAFFT v7 [17, 18] (alignment length 29 841 bp, 99.7 % pairwise identity). We generated a phylogenetic tree in IQ-TREE v2.0.6 [19] employing the HKY+G model of nucleotide substitution [20, 21] and used TempEst to explore the temporal signal of the dataset [22].

### Inferring importation events into Victoria via discrete trait analysis phylogeography

In our analyses, the locations explored consisted of the continents Africa, Europe, North America, Asia and South America (individual countries included in each deme are listed in Table S2). We have separated Asian sequences into Chinese sequences (PR China) and the remaining Asian sequences (Asia). Similarly, European sequences were split into UK sequences and the remaining European sequences (Europe). We chose to isolate PR China from Asia as a separate deme, as it is the country were SARS-CoV-2 was first detected [23]. We isolated the UK from Europe as a separate deme based on the large proportion of sequences with associated travel history listed as the UK (Fig. S1c). We also included sequences from Australia and New Zealand (NZ), as they are the two countries within Oceania that had a significant number of SARS-CoV-2 cases and were likely to contribute to the overall importation and exportation dynamics.

Firstly, we utilized a similar method to those used to infer geographical movement of SARS-CoV-2 between Nordic countries [24]. Our methodology set a geographical deme for each tip, based on the sampling location [24, 25]. We inferred the frequency of importation events into Victoria using a discrete trait analysis (DTA) via a discrete phylogeographical model known as Bayesian stochastic mapping approach. The posterior distribution generated ‘type-changes’ between locations along the branches. We utilized a coalescent exponential tree model, strict molecular clock and HKY+G substitution model. We ran a Markov chain Monte Carlo (MCMC) of 1×10^8^ chain length, sampled at every 10 000th step.

### Implementing travel history into phylogeography models to infer importation events into Victoria

To incorporate travel history metadata into a phylogeographical model, we modified previous methods [10]. As above, we set a geographical deme for each tip, based on the sampling location. In sequences with travel history metadata, we included the travel history location as an ancestral node (Fig. S2). During this time period, the incidence of COVID-19 in the Australian community was very low, hence the implicit assumption that COVID-19 cases in returned overseas travellers at this time are most likely to represent acquisition outside of Australia.We ran a MCMC, as above. For the DTA-TH analysis, all effective sample size of all parameters were >200 estimated in Tracer [26], indicating that sufficient sampling from the posterior was generated. To ensure the robustness of our model, we ran two replicates (Fig. S3).

### Assessing key travel routes of SARS-COV-2 importation into Australia and impacts of public health measures

To visualize the results from DTA-TH, we took the maximum *a posteriori* (MAP) trajectory, which captures the migration trajectory and associated parameters with the highest posterior support. The MAP trajectory coincides with a clearly defined mode in the posterior (Fig. S4), validating its use. We plotted the MAP of both replicates in R to analyse the proportion and temporal structure of importations and exportations (available at www.github.com/aporter704/phylogeo).

We assessed the impact of border closures on domestic outbreak risk (within Victoria), plotting information from the Australian Federal Government and the Victorian Government about the introduction of several travel bans, enforced quarantine and other public health measures [27].

## Results

As individual travel history was collected extensively in Victoria, using this information alone can be used to infer where the bulk of SARS-CoV-2 importations originated (Fig. S5). Based on reported travel history, we anticipated that many SARS-CoV-2-positive travellers were returning from North America, the UK and New Zealand (Fig. S1).

A critical finding from our analysis is that the implementation of travel history metadata into phylogeographical models is essential for accurate estimates of SARS-CoV-2 movement during the first-wave of COVID-19 in Australia. The DTA model was unable to reach completion of MCMC due to a likelihood underflow. Therefore, we do not report the results from this analysis. We hypothesize that in our DTA analysis there is insufficient estimation of the movement between states. This is demonstrated by the significant difference in non-zero rates estimated by DTA, compared to DTA-TH. This statistic (non-zero rates) is an indication of how many times a path between states is sampled in the MCMC. The DTA model had a mean of 8 (95 % HPD: 5, 12) non-zero rates, in comparison to the DTA-TH model, which had a mean of 22 (95 % HPD: 16, 29).

**Fig. 2.**
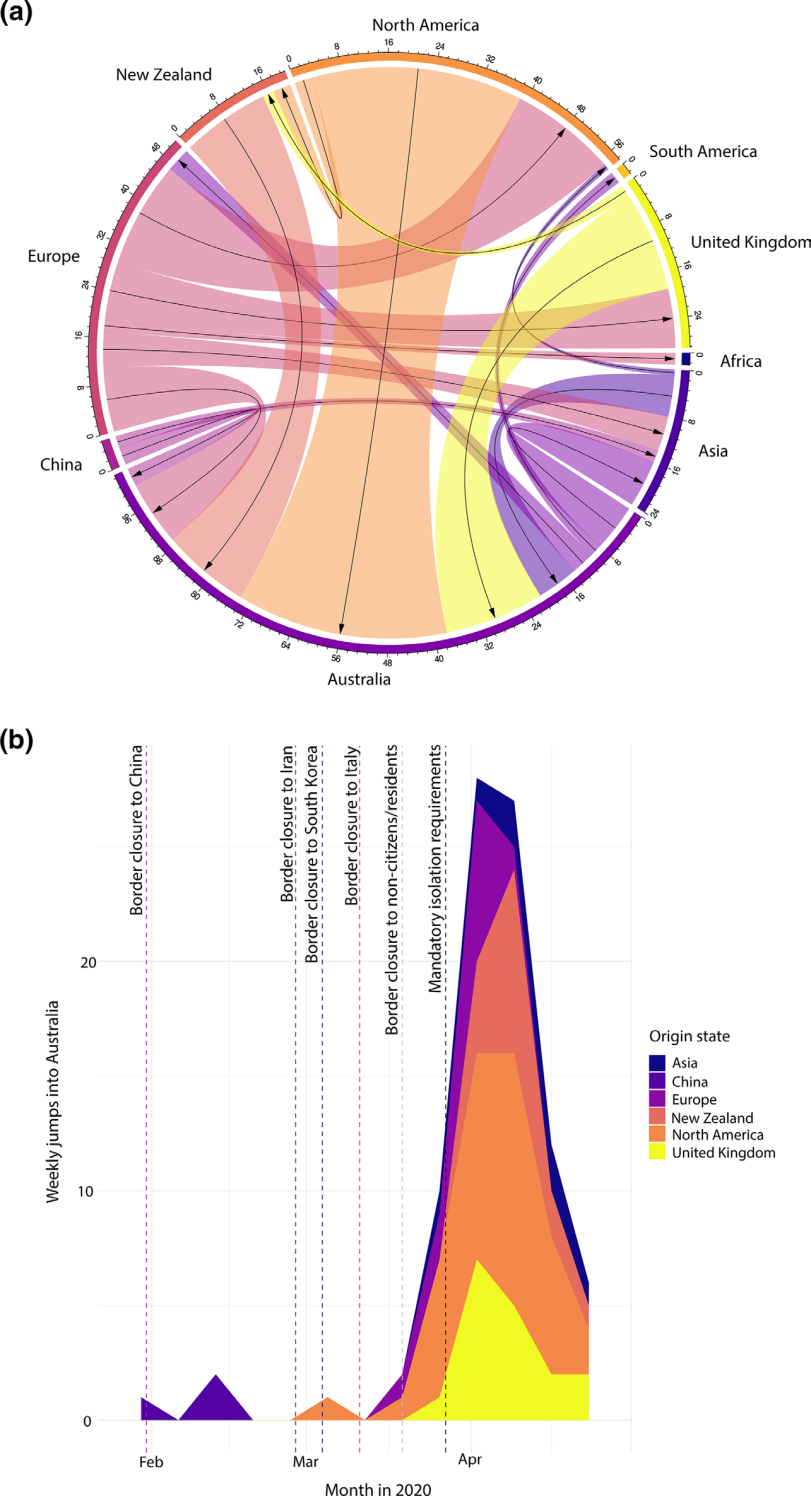
Visualization of SARS-CoV-2 importation and exportation estimates of the Australian first-wave generated using the DTA-TH methodology. (**a**) The directionality of exportations and importations to and from each deme, as estimated by the DTA-TH model. The size of the coloured bands represents the posterior median number of inferred migration events, and the black arrows represent the direction of migration. (**b**) The number of migrations into Australia as estimated by DTA-TH. To the right, the legend describes the colours representing the origin of importations, with Asia (blue), PR China (indigo), Europe (purple), North America (pink), New Zealand (dark orange) and the UK (yellow). The dotted lines represent the timing of the border restrictions enforced, from left to right: PR China (purple), Iran (indigo), Republic of Korea (blue) and Italy (magenta), the border closure against all non-citizens and residents (grey), and the introduction of Australia’s mandatory isolation requirements (black). The *x*-axis represents time in months of 2020 and the *y*-axis represents the number of Markov jumps into Australia (i.e. importations) per week.

Estimates from DTA-TH suggest that Australia was not a major exporter of the virus to global locations in this time (Fig. S5), and a substantial proportion of importations were coming from North America, followed by the UK and New Zealand (Fig. 2a).

The majority of SARS-CoV-2 importations occurred after the enforcement of travel bans and quarantine measures, during mid-March until mid-May 2020 (Fig. 2b), with a relatively lower proportion of exportations throughout the first wave (Fig. S5).

## Discussion

Our estimates from DTA-TH suggest that North America, the UK and New Zealand were major origins of importations of SARS-CoV-2 into Australia during this time period. This could be due to the high proportion of Australians residing in these countries (e.g. a third of the estimated number of Australians living overseas reside in the UK, approximately 200 000 people) [28], many of whom may have returned to Australia at the beginning of the COVID-19 pandemic.

Previous work [5, 13, 29] has discussed the impacts of the total border closure of Australia, 14 day isolation requirements and social distancing measures in March 2020, which, combined, may have made an impact on slowing the spread of SARS-CoV-2 in Australia (Fig. 1).

In our analysis, we can observe a slowing of international SARS-CoV-2 introductions into Australia mid-April 2020 (Fig. 2). Due to the multitude of measures that were co-occurring in the first wave (Fig. 1), it is difficult to determine the single factor (or the combination of factors) that had a significant impact on slowing the introductions into Australia. Additionally, we anticipate that there was likely a lag phase between the enforcement of a control measure (e.g. the border closure and mandatory isolation requirements in late March 2020) and the effect on virus introductions. For example, even though social distancing measures were introduced in March, cases of SARS-CoV-2 were still being imported after that date from international locations. These infections may have not led to community transmission, with the enforcement of hotel quarantine (Fig. 1). However, we do not have sufficient evidence to advise which element in Australia’s response had the strongest impact on reducing introductions, and furthermore, the best management strategies for preventing the spread of highly transmissible viruses, including SARS-CoV-2, remains a controversial topic [30].

The marked difference between the DTA-TH and DTA models is the inability of the DTA to reach completion of MCMC runs. This suggests that, for the analysis of SARS-CoV-2 movement in the first wave of COVID-19 in Australia, the use of travel history metadata is essential to generate estimates. Importantly, the results from the DTA-TH model suggest that Australia was not a major exporter of SARS-CoV-2 to other locations in the first wave of COVID-19 (Fig. S5). Although the DTA MCMC did not converge to a stationary distribution, we anticipate that estimates generated by a model lacking travel history metadata would be biased towards Australia playing a major role in the exportation of the virus, due to the high proportion of sequencing in Australia compared to other locations (Table 1, Fig. S1). Although we cannot measure the difference in estimates between the two models we have analysed, we highlight that applying phylogeography models to biased datasets without the inclusion of metadata can either reduce the ability of standard DTA models to converge, or potentially lead to misinformed estimates.

We assume that migrations between the global demes have not been fully resolved in this analysis. The dataset was curated specifically for focusing on the importation and exportation of Australian sequences, to examine the bias we assumed was present in the global dataset due to the high sampling frequency during the first-wave of COVID-19. We acknowledge that our global sequence dataset (*n*=200) only provides a snapshot of the movement of SARS-CoV-2 internationally.

We emphasize the importance of the inclusion of metadata within phylogeographical frameworks and recommend caution when applying DTA models without travel history to estimate the spatiotemporal movement of SARS-CoV-2 and other viruses from datasets that have sampling bias present. Including additional streams of metadata into phylogeographical models can not only help alleviate sampling bias, but in certain cases could be essential to generate accurate estimates. This is particularly critical when considering the SARS-CoV-2 global dataset as many locations, including Australia, have high sampling frequencies.

## Conclusions

The presence of sampling bias within the global dataset of SARS-CoV-2 sequences has previously been established, with the majority of SARS-CoV-2 genomes originating from high-income countries. The degree of bias made the dataset incompatible with standard phylogeographical methods, leading to innovative methodology to reduce bias and improve estimates [10].

Previous work has called for the optimization of data collection for downstream phylogenomic analysis, such as phylogeographical models, as estimates generated from these analyses have played a key role in a ‘genomics-informed response’ to the COVID-19 pandemic in real time [4].

This study exemplifies the importance of the inclusion of metadata in phylogeographical modelling when exploring datasets with known bias. Based on the number of Australian sequences alone (Table 1), it could be postulated that Australia played a role in the global exportation of SARS-CoV-2 during the first wave of COVID-19. Although the DTA analysis did not converge, we would presume that, based on this sequencing bias, Australia would appear to be a major exporter of SARS-CoV-2 to other locations. However, with the addition of travel history metadata, we demonstrate that Australia was not a major exporter of SARS-CoV-2 to other locations.

We emphasize the importance of the inclusion of metadata within phylogeographical frameworks and recommend caution when applying phylogeographical models without travel history to estimate the global spatiotemporal movement of SARS-CoV-2. Our results suggest that the inclusion of travel history metadata can aid in the alleviation of the sampling bias present in the global SARS-CoV-2 dataset. Specifically, in this analysis, the implementation of travel history metadata was essential for generating accurate estimates.

This study further highlights the importance of collecting and sharing metadata to support the ongoing COVID-19 pandemic response [4], along with future infectious disease outbreak scenarios.

## Supplementary Data

Supplementary material 1
