# Supplementary material for: The importance of utilizing travel history metadata for informative phylogeographical inferences: a case study of early SARS-CoV-2 introductions into Australia
Source: Microb Genom. 2023 Aug 31;9(8):mgen001099. doi: 10.1099/mgen.0.001099 (PMC10483412; doi:10.1099/mgen.0.001099)
Supplement: Supplementary material 1 [file mgen-9-1099-s001.pdf]

Supplementary Materials

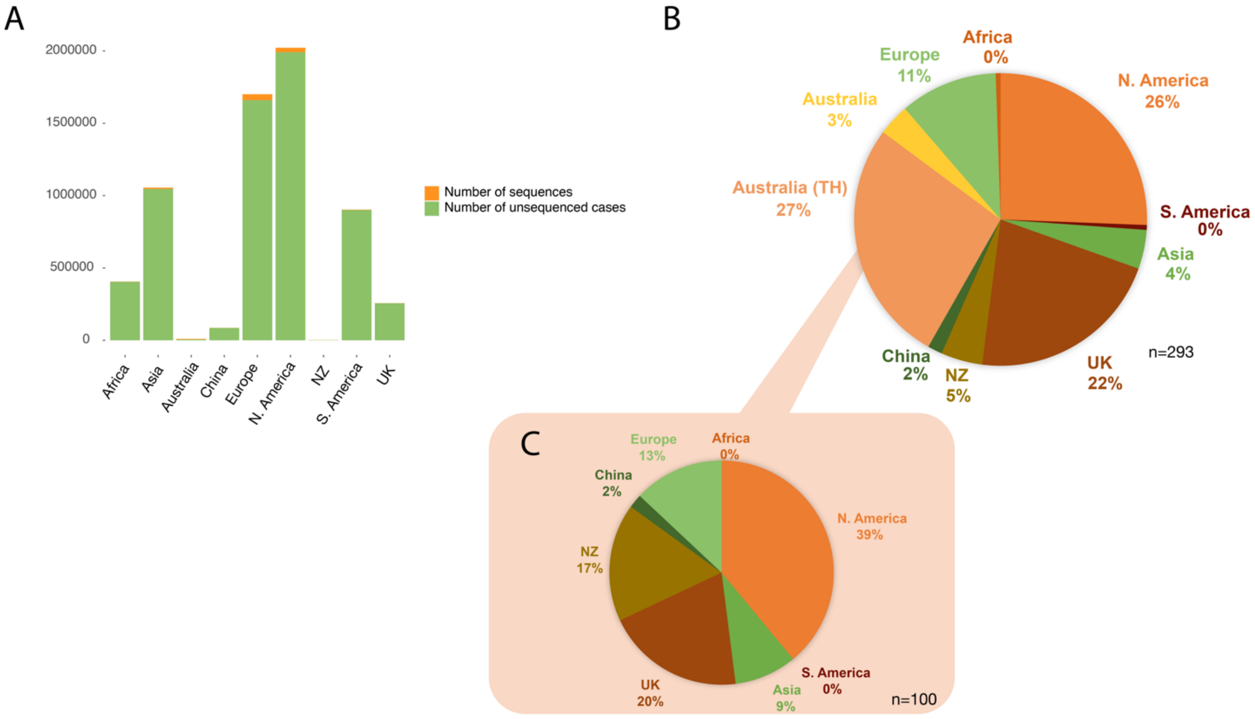

Supplementary Figure S1. An overview of SARS-CoV-2 sequences and samples from the first wave of COVID-19. **(A)** A visualisation of the proportion of reported COVID-19 cases (sourced from ourworldofdata.com) from the first wave (January-May 2020) that have SARS-COV-2 sequences in GISAID. The total proportion of cases and available sequences are plotted on the y-axis, with each geographical deme on the x-axis. Cases that were not sequenced are shown in green (bottom), and the proportion of available sequences are shown in orange (top), based on data in Table 1. **(B)** A pie chart of the location of sampling reported for the total number of global sequences used in the analysis (n=293), from demes Europe, Africa, North America (N. America), South America (S. America), Asia, United Kingdom (UK), New Zealand (NZ), China, Australia, and Australia with travel history (TH). **(C)** A pie chart of location of recent travel history reported for the sequences from Victoria, Australia, that had individual travel history data available (n=100).

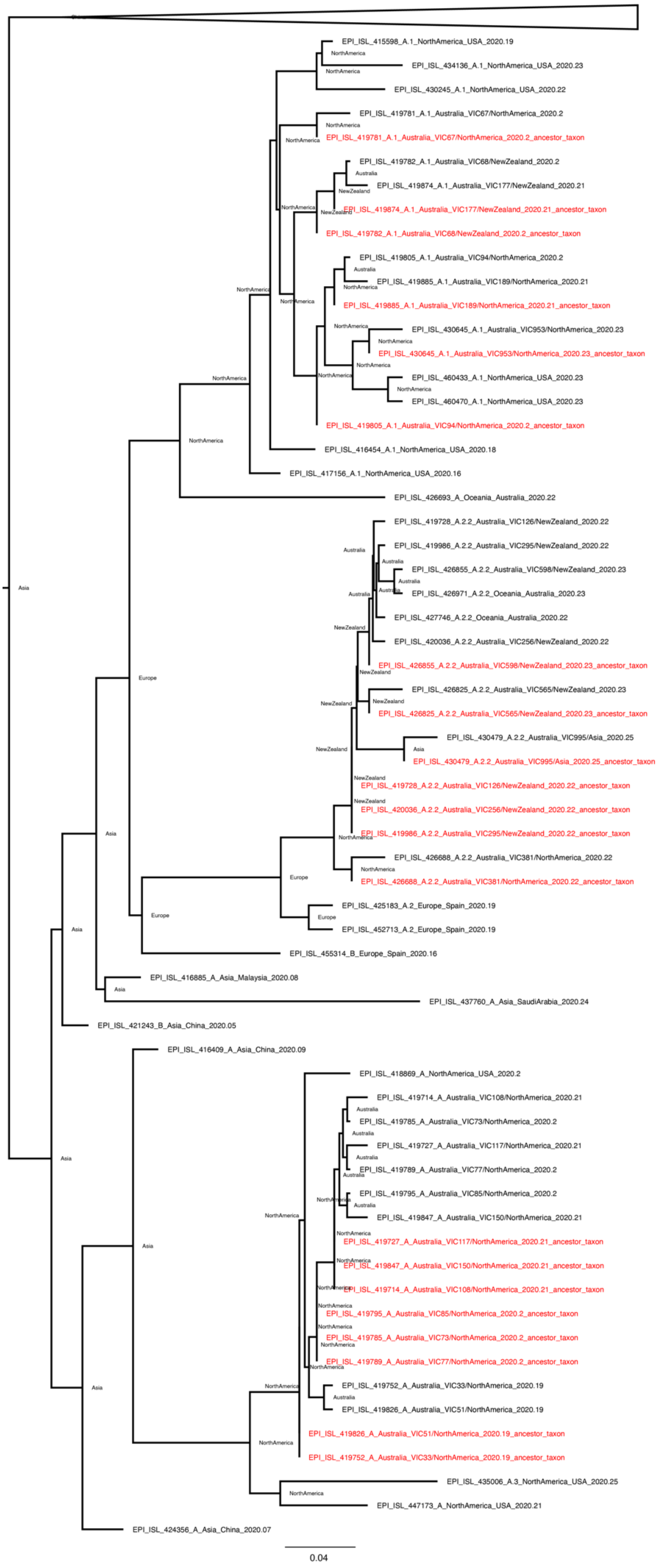
EPI_ISL_415598_A.1_NorthAmerica_USA_2020.19
EPI_ISL_434136_A.1_NorthAmerica_USA_2020.23
EPI_ISL_430245_A.1_NorthAmerica_USA_2020.22
EPI_ISL_419781_A.1_Australia_VIC67/NorthAmerica_2020.2
EPI_ISL_419781_A.1_Australia_VIC67/NorthAmerica_2020.2_ancestor_taxon
EPI_ISL_419782_A.1_Australia_VIC68/NewZealand_2020.2
EPI_ISL_419874_A.1_Australia_VIC177/NewZealand_2020.21
EPI_ISL_419874_A.1_Australia_VIC177/NewZealand_2020.21_ancestor_taxon
EPI_ISL_419782_A.1_Australia_VIC68/NewZealand_2020.2_ancestor_taxon
EPI_ISL_419805_A.1_Australia_VIC94/NorthAmerica_2020.2
EPI_ISL_419885_A.1_Australia_VIC189/NorthAmerica_2020.21
EPI_ISL_419885_A.1_Australia_VIC189/NorthAmerica_2020.21_ancestor_taxon
EPI_ISL_430645_A.1_Australia_VIC953/NorthAmerica_2020.23
EPI_ISL_430645_A.1_Australia_VIC953/NorthAmerica_2020.23_ancestor_taxon
EPI_ISL_460433_A.1_NorthAmerica_USA_2020.23
EPI_ISL_460470_A.1_NorthAmerica_USA_2020.23
EPI_ISL_419805_A.1_Australia_VIC94/NorthAmerica_2020.2_ancestor_taxon
EPI_ISL_416454_A.1_NorthAmerica_USA_2020.18
EPI_ISL_417156_A.1_NorthAmerica_USA_2020.16
EPI_ISL_426693_A_Oceania_Australia_2020.22
EPI_ISL_419728_A.2.2_Australia_VIC126/NewZealand_2020.22
EPI_ISL_419986_A.2.2_Australia_VIC295/NewZealand_2020.22
EPI_ISL_426855_A.2.2_Australia_VIC598/NewZealand_2020.23
EPI_ISL_426971_A.2.2_Oceania_Australia_2020.23
EPI_ISL_427746_A.2.2_Oceania_Australia_2020.22
EPI_ISL_420036_A.2.2_Australia_VIC256/NewZealand_2020.22
EPI_ISL_426855_A.2.2_Australia_VIC598/NewZealand_2020.23_ancestor_taxon
EPI_ISL_426825_A.2.2_Australia_VIC565/NewZealand_2020.23
EPI_ISL_426825_A.2.2_Australia_VIC565/NewZealand_2020.23_ancestor_taxon
EPI_ISL_430479_A.2.2_Australia_VIC995/Asia_2020.25
EPI_ISL_430479_A.2.2_Australia_VIC995/Asia_2020.25_ancestor_taxon
EPI_ISL_419728_A.2.2_Australia_VIC126/NewZealand_2020.22_ancestor_taxon
EPI_ISL_420036_A.2.2_Australia_VIC256/NewZealand_2020.22_ancestor_taxon
EPI_ISL_419986_A.2.2_Australia_VIC295/NewZealand_2020.22_ancestor_taxon
EPI_ISL_426688_A.2.2_Australia_VIC381/NorthAmerica_2020.22
EPI_ISL_426688_A.2.2_Australia_VIC381/NorthAmerica_2020.22_ancestor_taxon
EPI_ISL_425183_A.2_Europe_Spain_2020.19
EPI_ISL_452713_A.2_Europe_Spain_2020.19
EPI_ISL_455314_B_Europe_Spain_2020.16
EPI_ISL_416885_A_Asia_Malaysia_2020.08
EPI_ISL_437760_A_Asia_SaudiArabia_2020.24
EPI_ISL_421243_B_Asia_China_2020.05
EPI_ISL_416409_A_Asia_China_2020.09
EPI_ISL_418869_A_NorthAmerica_USA_2020.2
EPI_ISL_419714_A_Australia_VIC108/NorthAmerica_2020.21
EPI_ISL_419785_A_Australia_VIC73/NorthAmerica_2020.2
EPI_ISL_419727_A_Australia_VIC117/NorthAmerica_2020.21
EPI_ISL_419789_A_Australia_VIC77/NorthAmerica_2020.2
EPI_ISL_419795_A_Australia_VIC85/NorthAmerica_2020.2
EPI_ISL_419847_A_Australia_VIC150/NorthAmerica_2020.21
EPI_ISL_419727_A_Australia_VIC117/NorthAmerica_2020.21_ancestor_taxon
EPI_ISL_419847_A_Australia_VIC150/NorthAmerica_2020.21_ancestor_taxon
EPI_ISL_419714_A_Australia_VIC108/NorthAmerica_2020.21_ancestor_taxon
EPI_ISL_419795_A_Australia_VIC85/NorthAmerica_2020.2_ancestor_taxon
EPI_ISL_419785_A_Australia_VIC73/NorthAmerica_2020.2_ancestor_taxon
EPI_ISL_419789_A_Australia_VIC77/NorthAmerica_2020.2_ancestor_taxon
EPI_ISL_419752_A_Australia_VIC33/NorthAmerica_2020.19
EPI_ISL_419826_A_Australia_VIC51/NorthAmerica_2020.19
EPI_ISL_419826_A_Australia_VIC51/NorthAmerica_2020.19_ancestor_taxon
EPI_ISL_419752_A_Australia_VIC33/NorthAmerica_2020.19_ancestor_taxon
EPI_ISL_435006_A.3_NorthAmerica_USA_2020.25
EPI_ISL_447173_A_NorthAmerica_USA_2020.21
EPI_ISL_424356_A_Asia_China_2020.07
0.04

Supplementary Figure S2. A subset of the full maximum clade credibility (MCC) tree of the DTA-TH analysis. The MCC tree for DTA-TH (replicate 1) with the most supported ancestral deme listed at each node shown in this subset, and with each ancestral taxon for Australian sequences with travel history highlighted in red. The scale bar represents substitutions/site/year.

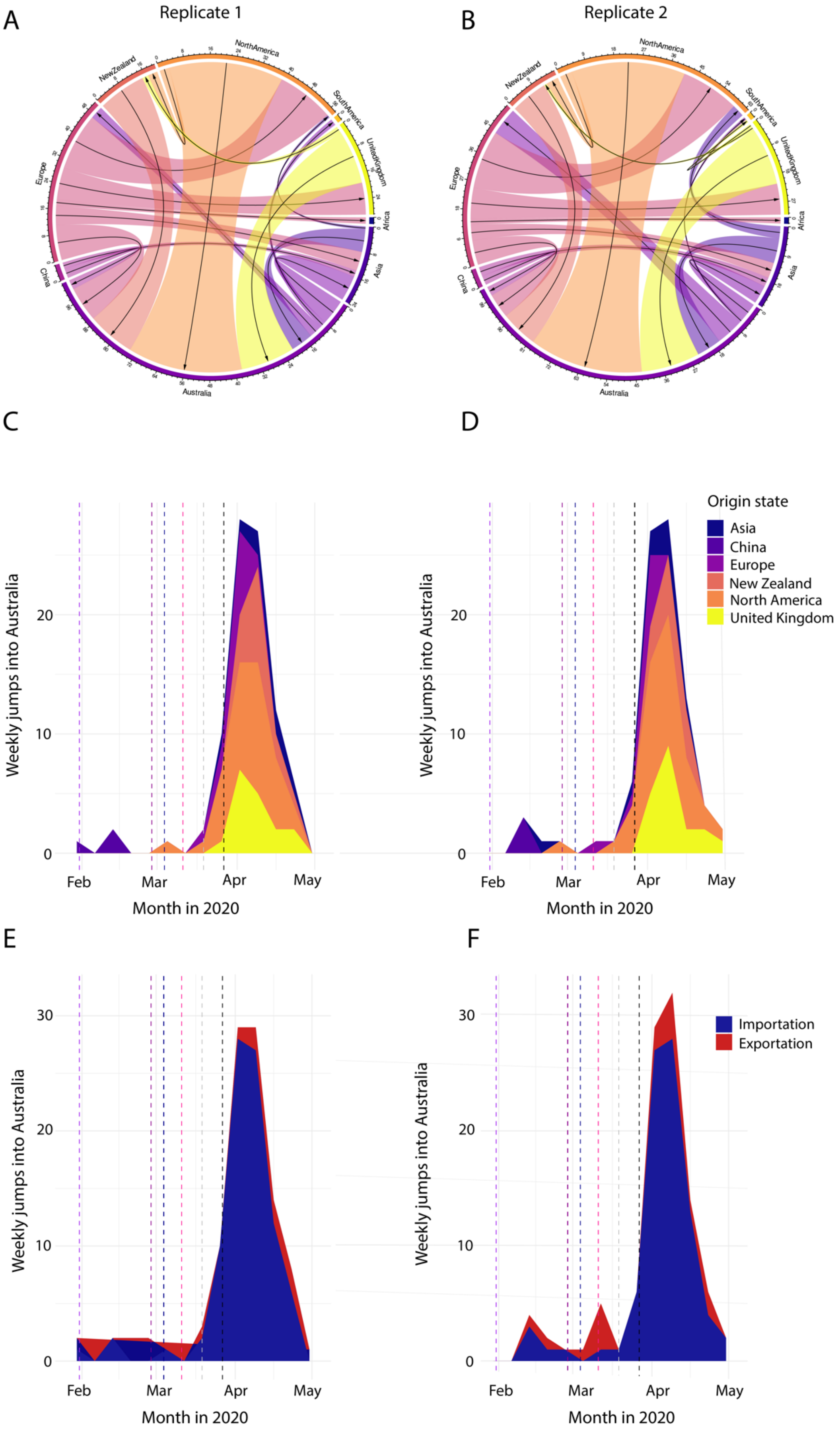
A
Replicate 1
B
Replicate 2
NorthAmerica
NewZealand
SouthAmerica
UnitedKingdom
Africa
Asia
Australia
China
Europe
C
D
Origin state
Asia
China
Europe
New Zealand
North America
United Kingdom
Weekly jumps into Australia
Month in 2020
Feb
Mar
Apr
May
E
F
Importation
Exportation

Supplementary Figure S3. A comparison of the two independent replicates of DTA-TH. **(A,B)** The directionality of exportations and importations to and from each deme, as estimated by the DTA-TH model. The size of the coloured bands represents the posterior median number of inferred migration events, and the black arrows represent the direction of migration. **(C,D)** The number of migrations into Australia as estimated by DTA-TH. To the right, the legend describes the colours representing the origin of importations, with Asia (blue), China (indigo), Europe (purple), North America (pink), New Zealand (dark orange), and the UK (yellow). The dotted lines represent the timing of the border restrictions enforced, from left to right: China (purple), Iran (indigo), South Korea (blue) and Italy (magenta), the border closure against all non-citizens and residents (grey), and the introduction of Australia's mandatory isolation requirements (black). The x-axis represents time in months of 2020, and y-axis represents the number of Markov jumps into Australia per week. **(E,F)** The number of importations into Australia (blue) and exportations out of Australia (red), as estimated by the DTA-TH model. The dotted lines represent the timing of the border restrictions enforced, from left to right: China (purple), Iran (indigo), South Korea (blue) and Italy (magenta), the border closure against all non-citizens and residents (grey), and the introduction of Australia's mandatory isolation requirements (black). The x-axis represents time in months of 2020, and y-axis represents the number of Markov jumps per week.

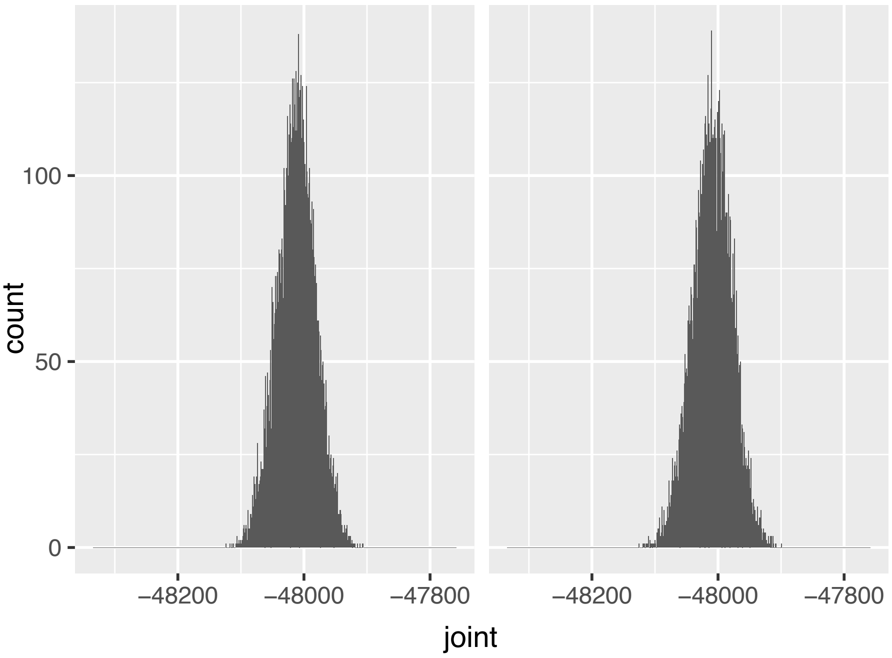

Supplementary Figure S4. A visualisation of the combined probability distribution of the model, parameters, and migration trajectory for each step of the iterative MCMC, for both replicates of DTA-TH. Importantly, the two replicates both have a clearly defined mode, from which the MAP trajectory is drawn.

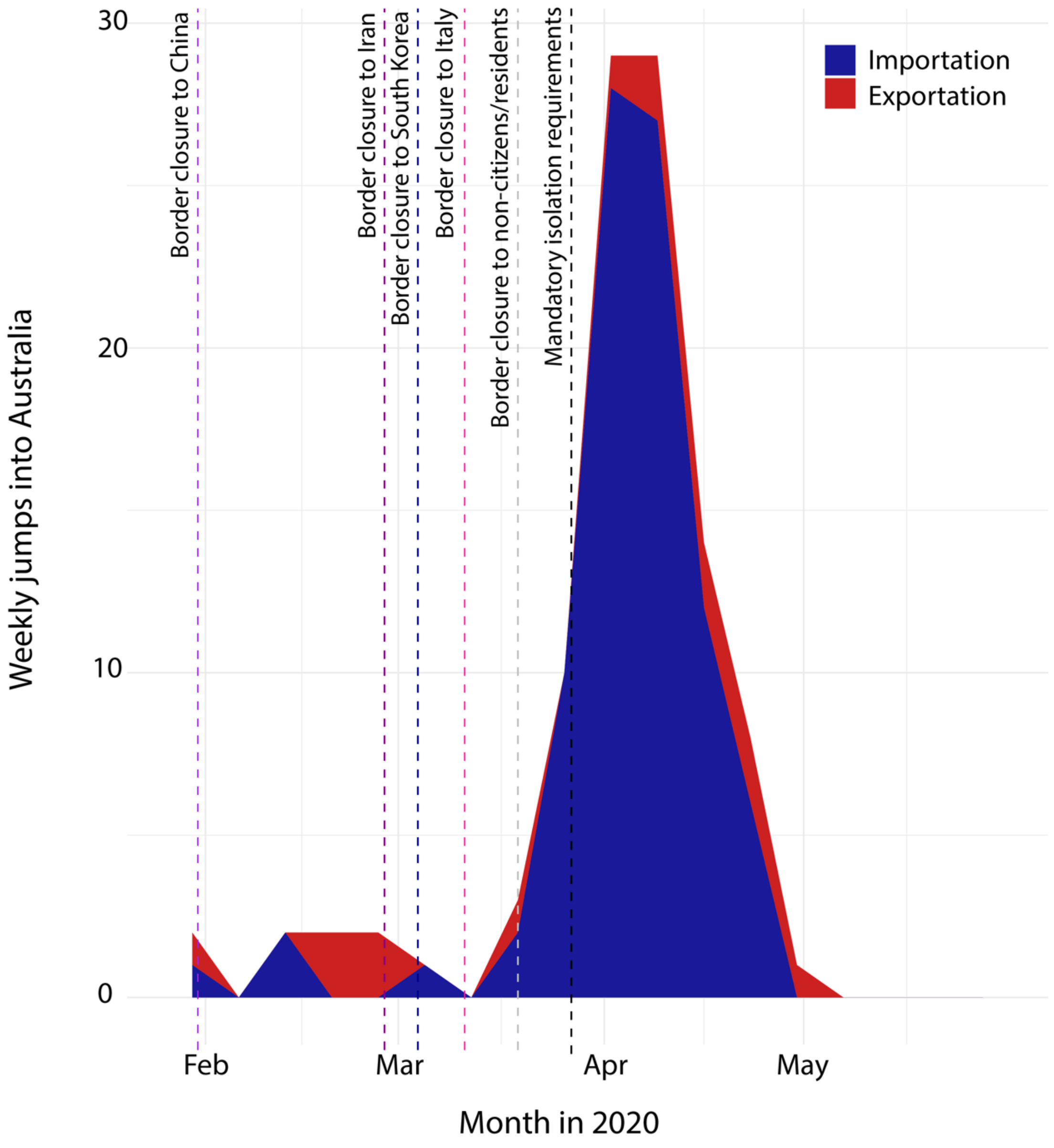

Supplementary Figure S5. The number of importations into Australia (blue) and exportations out of Australia (red), as estimated by the DTA-TH model. The dotted lines represent the timing of the border restrictions enforced, from left to right: China (purple), Iran (indigo), South Korea (blue) and Italy (magenta), the border closure against all non-citizens and residents (grey), and the introduction of Australia's mandatory isolation requirements (black). The x-axis represents time in months of 2020, and y-axis represents the number of Markov jumps per week.

Supplementary Table 1. Individual accession numbers for GISAID sequences used in this study.

Accession	Lineage	Deme	Country of sampling	Date (decimal)
**EPI_ISL_412975**	B.4	Australia	Australia	2020.16
**EPI_ISL_427746**	A.2.2	Australia	Australia	2020.22
**EPI_ISL_451595**	B.4.4	Australia	Australia	2020.22
**EPI_ISL_451619**	B.1	Australia	Australia	2020.28
**EPI_ISL_427773**	B.4.6	Australia	Australia	2020.22
**EPI_ISL_420011**	B.1.23	Australia	Australia	2020.23
**EPI_ISL_426693**	A	Australia	Australia	2020.22
**EPI_ISL_426781**	B.31	Australia	Australia	2020.22
**EPI_ISL_426782**	B.1.1	Australia	Australia	2020.22
**EPI_ISL_426971**	A.2.2	Australia	Australia	2020.23
**EPI_ISL_426977**	B.1	Australia	Australia	2020.24
**EPI_ISL_427025**	B.4.5	Australia	Australia	2020.25
**EPI_ISL_430545**	B.1.23	Australia	Australia	2020.24
**EPI_ISL_438103**	B.1.8	Europe	Austria	2020.23
**EPI_ISL_437203**	B	Europe	Austria	2020.26
**EPI_ISL_424356**	A	China	China	2020.07
**EPI_ISL_451935**	B.1	Europe	Belgium	2020.21
**EPI_ISL_416467**	B.1.1	Europe	Belgium	2020.17
**EPI_ISL_450307**	B.1	North America	Canada	2020.23
**EPI_ISL_447816**	B.1	South America	Colombia	2020.27
**EPI_ISL_447781**	B.1.420	South America	Colombia	2020.25
**EPI_ISL_426890**	B.1	Europe	Czech Republic	2020.23
**EPI_ISL_429277**	B.1.510	Europe	Denmark	2020.19
**EPI_ISL_437001**	B.1.1.70	Europe	Denmark	2020.23
**EPI_ISL_437031**	B.1.118	Europe	Denmark	2020.23
**EPI_ISL_429431**	B.1.343	Europe	Denmark	2020.19
**EPI_ISL_429464**	B.1	Europe	Denmark	2020.22
**EPI_ISL_416144**	B.1	Europe	Denmark	2020.16
**EPI_ISL_437356**	B.1	Africa	Democratic Republic of the Congo	2020.3
**EPI_ISL_423938**	B.40	United Kingdom	United Kingdom	2020.21
**EPI_ISL_423144**	B.1.1	United Kingdom	United Kingdom	2020.23
**EPI_ISL_423244**	B.1.1.89	United Kingdom	United Kingdom	2020.23
**EPI_ISL_421879**	B.1.1	United Kingdom	United Kingdom	2020.23
**EPI_ISL_423435**	B.1	United Kingdom	United Kingdom	2020.24
**EPI_ISL_423481**	B.1.1	United Kingdom	United Kingdom	2020.25
**EPI_ISL_444285**	B.1.1	United Kingdom	United Kingdom	2020.29
**EPI_ISL_442729**	B.1.1	United Kingdom	United Kingdom	2020.27
**EPI_ISL_442764**	B.1.1.372	United Kingdom	United Kingdom	2020.27
**EPI_ISL_442706**	B.1	United Kingdom	United Kingdom	2020.27
**EPI_ISL_443717**	B.1	United Kingdom	United Kingdom	2020.23
**EPI_ISL_444384**	B.1.1	United Kingdom	United Kingdom	2020.32

**EPI_ISL_439656**	B.10	United Kingdom	United Kingdom	2020.24
**EPI_ISL_438299**	B.3	United Kingdom	United Kingdom	2020.24
**EPI_ISL_438315**	B.1.1	United Kingdom	United Kingdom	2020.24
**EPI_ISL_438309**	B.28	United Kingdom	United Kingdom	2020.24
**EPI_ISL_439424**	B.1.1	United Kingdom	United Kingdom	2020.25
**EPI_ISL_441125**	B.1.1	United Kingdom	United Kingdom	2020.25
**EPI_ISL_441158**	B.1.1	United Kingdom	United Kingdom	2020.25
**EPI_ISL_441269**	B.40	United Kingdom	United Kingdom	2020.25
**EPI_ISL_433828**	B.1.1	United Kingdom	United Kingdom	2020.27
**EPI_ISL_433829**	B.40	United Kingdom	United Kingdom	2020.27
**EPI_ISL_433928**	B.1.1	United Kingdom	United Kingdom	2020.28
**EPI_ISL_441564**	B.1.1	United Kingdom	United Kingdom	2020.26
**EPI_ISL_442338**	B	United Kingdom	United Kingdom	2020.26
**EPI_ISL_438590**	B	United Kingdom	United Kingdom	2020.31
**EPI_ISL_443545**	B.1.391	United Kingdom	United Kingdom	2020.28
**EPI_ISL_438701**	B.1.1	United Kingdom	United Kingdom	2020.32
**EPI_ISL_439788**	B.1	United Kingdom	United Kingdom	2020.28
**EPI_ISL_449654**	B.1.1.254	United Kingdom	United Kingdom	2020.26
**EPI_ISL_444134**	B.1	United Kingdom	United Kingdom	2020.27
**EPI_ISL_444162**	B.1.1	United Kingdom	United Kingdom	2020.27
**EPI_ISL_448980**	B.1.1	United Kingdom	United Kingdom	2020.3
**EPI_ISL_449017**	B.1	United Kingdom	United Kingdom	2020.3
**EPI_ISL_449035**	B.1.1	United Kingdom	United Kingdom	2020.34
**EPI_ISL_449109**	B.1.1	United Kingdom	United Kingdom	2020.27
**EPI_ISL_448299**	B.1.93	United Kingdom	United Kingdom	2020.29
**EPI_ISL_448369**	B.1.1	United Kingdom	United Kingdom	2020.34
**EPI_ISL_448457**	B.1.1	United Kingdom	United Kingdom	2020.27
**EPI_ISL_448787**	B.1.465	United Kingdom	United Kingdom	2020.27
**EPI_ISL_420240**	B.1.1.369	United Kingdom	United Kingdom	2020.22
**EPI_ISL_441975**	B.1.1	United Kingdom	United Kingdom	2020.23
**EPI_ISL_442370**	B.1	United Kingdom	United Kingdom	2020.27
**EPI_ISL_453746**	B.1.1	United Kingdom	United Kingdom	2020.31
**EPI_ISL_453759**	B.1	United Kingdom	United Kingdom	2020.3
**EPI_ISL_432622**	B.1.1	United Kingdom	United Kingdom	2020.25
**EPI_ISL_432642**	B.1.1	United Kingdom	United Kingdom	2020.25
**EPI_ISL_418404**	B.1.465	Europe	Finland	2020.2
**EPI_ISL_428348**	B.1.212	Europe	France	2020.22
**EPI_ISL_426059**	B.1	Europe	Germany	2020.2
**EPI_ISL_417536**	B.1	Europe	Iceland	2020.2
**EPI_ISL_417619**	B.1.1	Europe	Iceland	2020.21
**EPI_ISL_424435**	B.40	Europe	Iceland	2020.21
**EPI_ISL_417675**	B.1	Europe	Iceland	2020.19
**EPI_ISL_450784**	B.6	Asia	India	2020.35

**EPI_ISL_447863**	B.6	Asia	India	2020.28
**EPI_ISL_447450**	B.1.1.50	Asia	Israel	2020.24
**EPI_ISL_447384**	B.1	Asia	Israel	2020.25
**EPI_ISL_447324**	B.36	Asia	Israel	2020.21
**EPI_ISL_447280**	B.1	Asia	Israel	2020.23
**EPI_ISL_451306**	B.1	Europe	Italy	2020.14
**EPI_ISL_434516**	B.40	Asia	Jordan	2020.2
**EPI_ISL_437093**	B.1.1	Europe	Latvia	2020.22
**EPI_ISL_416885**	A	Asia	Malaysia	2020.08
**EPI_ISL_421243**	B	China	China	2020.05
**EPI_ISL_455123**	B.1.22	Europe	Netherlands	2020.19
**EPI_ISL_422684**	B	Europe	Netherlands	2020.21
**EPI_ISL_422919**	B.11	Europe	Netherlands	2020.21
**EPI_ISL_455448**	B.1.1	Europe	Poland	2020.25
**EPI_ISL_418007**	B.1.1	Europe	Portugal	2020.2
**EPI_ISL_418008**	B.1.1	Europe	Portugal	2020.2
**EPI_ISL_421457**	B.3	Europe	Portugal	2020.21
**EPI_ISL_421497**	B.1.1	Europe	Portugal	2020.23
**EPI_ISL_454089**	B.1	Europe	Portugal	2020.21
**EPI_ISL_454133**	B.1.1	Europe	Portugal	2020.22
**EPI_ISL_445220**	B.1.1	Europe	Romania	2020.33
**EPI_ISL_455469**	B.1.408	Europe	Romania	2020.36
**EPI_ISL_428862**	B.1.1	Europe	Russia	2020.19
**EPI_ISL_436715**	B.1.1	Europe	Russia	2020.28
**EPI_ISL_436717**	B.1.1	Europe	Russia	2020.28
**EPI_ISL_450265**	B.1.1	Europe	Russia	2020.3
**EPI_ISL_428921**	B.1	Europe	Russia	2020.21
**EPI_ISL_437760**	A	Asia	Saudi Arabia	2020.24
**EPI_ISL_433595**	B	United Kingdom	United Kingdom	2020.25
**EPI_ISL_425724**	B.1	United Kingdom	United Kingdom	2020.19
**EPI_ISL_438910**	B.1.93	United Kingdom	United Kingdom	2020.28
**EPI_ISL_425875**	B.1.1	United Kingdom	United Kingdom	2020.22
**EPI_ISL_433374**	B.1.391	United Kingdom	United Kingdom	2020.26
**EPI_ISL_439188**	B.40	United Kingdom	United Kingdom	2020.31
**EPI_ISL_439207**	B.1	United Kingdom	United Kingdom	2020.31
**EPI_ISL_439261**	B.1	United Kingdom	United Kingdom	2020.32
**EPI_ISL_439347**	B.1	United Kingdom	United Kingdom	2020.33
**EPI_ISL_418207**	B.1.356	Africa	Senegal	2020.17
**EPI_ISL_416409**	A	China	China	2020.09
**EPI_ISL_452571**	B.1	Europe	Spain	2020.23
**EPI_ISL_455314**	B	Europe	Spain	2020.16
**EPI_ISL_430719**	B	Europe	Spain	2020.19
**EPI_ISL_452713**	A.2	Europe	Spain	2020.19

**EPI_ISL_447485**	B.1	Europe	Spain	2020.24
**EPI_ISL_425183**	A.2	Europe	Spain	2020.19
**EPI_ISL_454440**	B.1	Europe	Sweden	2020.19
**EPI_ISL_451719**	B.1	Europe	Switzerland	2020.2
**EPI_ISL_451814**	B.1	Europe	Switzerland	2020.25
**EPI_ISL_451835**	B.1.1	Europe	Switzerland	2020.25
**EPI_ISL_426530**	B.1.320	North America	USA	2020.21
**EPI_ISL_436656**	B.1.14	North America	USA	2020.27
**EPI_ISL_417931**	B	North America	USA	2020.21
**EPI_ISL_447589**	B.1.595	North America	USA	2020.23
**EPI_ISL_451278**	B.1	North America	USA	2020.34
**EPI_ISL_445130**	B.1.595	North America	USA	2020.25
**EPI_ISL_450231**	B.1	North America	USA	2020.25
**EPI_ISL_450177**	B.1	North America	USA	2020.27
**EPI_ISL_460126**	B.1	North America	USA	2020.23
**EPI_ISL_460470**	A.1	North America	USA	2020.23
**EPI_ISL_460433**	A.1	North America	USA	2020.23
**EPI_ISL_460373**	B.1.268	North America	USA	2020.26
**EPI_ISL_460265**	B.1	North America	USA	2020.25
**EPI_ISL_460110**	B.1	North America	USA	2020.23
**EPI_ISL_460129**	B.1	North America	USA	2020.24
**EPI_ISL_438225**	B.40	North America	USA	2020.22
**EPI_ISL_447173**	A	North America	USA	2020.21
**EPI_ISL_447221**	B.1	North America	USA	2020.21
**EPI_ISL_447059**	B.1.119	North America	USA	2020.2
**EPI_ISL_447098**	B.1.360	North America	USA	2020.25
**EPI_ISL_452284**	B.1.360	North America	USA	2020.33
**EPI_ISL_424901**	B.1	North America	USA	2020.17
**EPI_ISL_421712**	B.1.1	North America	USA	2020.21
**EPI_ISL_427485**	B.1	North America	USA	2020.25
**EPI_ISL_430340**	B.1	North America	USA	2020.24
**EPI_ISL_430353**	B.1.332	North America	USA	2020.25
**EPI_ISL_430415**	B.1	North America	USA	2020.27
**EPI_ISL_430430**	B.1.319	North America	USA	2020.28
**EPI_ISL_435540**	B.1	North America	USA	2020.3
**EPI_ISL_421623**	B.1	North America	USA	2020.22
**EPI_ISL_421633**	B	North America	USA	2020.21
**EPI_ISL_422515**	B.1	North America	USA	2020.22
**EPI_ISL_450010**	B.1	North America	USA	2020.22
**EPI_ISL_427607**	B.1	North America	USA	2020.2
**EPI_ISL_436485**	B.1	North America	USA	2020.23
**EPI_ISL_434908**	B.1	North America	USA	2020.23
**EPI_ISL_435000**	B.1	North America	USA	2020.25

**EPI_ISL_435006**	A.3	North America	USA	2020.25
**EPI_ISL_452154**	B.1.137	North America	USA	2020.27
**EPI_ISL_420019**	B.1	North America	USA	2020.21
**EPI_ISL_452815**	B.1	North America	USA	2020.27
**EPI_ISL_417156**	A.1	North America	USA	2020.16
**EPI_ISL_430245**	A.1	North America	USA	2020.22
**EPI_ISL_430261**	B.1.320	North America	USA	2020.22
**EPI_ISL_434136**	A.1	North America	USA	2020.23
**EPI_ISL_434320**	B.1	North America	USA	2020.23
**EPI_ISL_424221**	B.1.371	North America	USA	2020.22
**EPI_ISL_426100**	B.1.320	North America	USA	2020.23
**EPI_ISL_418869**	A	North America	USA	2020.2
**EPI_ISL_416454**	A.1	North America	USA	2020.18
**EPI_ISL_415625**	B.1.371	North America	USA	2020.19
**EPI_ISL_415598**	A.1	North America	USA	2020.19
**EPI_ISL_416492**	B.1	North America	USA	2020.2
**EPI_ISL_428301**	B.1.162	North America	USA	2020.23
**EPI_ISL_436631**	B.1	North America	USA	2020.23
**EPI_ISL_422053**	B.23	United Kingdom	United Kingdom	2020.23
**EPI_ISL_422178**	B.23	United Kingdom	United Kingdom	2020.24
**EPI_ISL_432390**	B.1.1.10	United Kingdom	United Kingdom	2020.24
**EPI_ISL_445617**	B.1	United Kingdom	United Kingdom	2020.27
**EPI_ISL_454921**	B	China	China	2020.14
**EPI_ISL_419841**	B.1.1	Australia	Australia	2020.21
**EPI_ISL_426683**	B.1.320	Australia	Australia	2020.22
**EPI_ISL_416413**	A.1	Australia	Australia	2020.17
**EPI_ISL_419796**	A	Australia	Australia	2020.2
**EPI_ISL_419732**	B	Australia	Australia	2020.22
**EPI_ISL_419885**	A.1	Australia	Australia	2020.21
**EPI_ISL_419935**	B.1.1	Australia	Australia	2020.22
**EPI_ISL_419902**	B.1	Australia	Australia	2020.21
**EPI_ISL_419805**	A.1	Australia	Australia	2020.2
**EPI_ISL_419937**	B	Australia	Australia	2020.22
**EPI_ISL_427095**	B.1.319	Australia	Australia	2020.25
**EPI_ISL_419864**	B.1.320	Australia	Australia	2020.21
**EPI_ISL_419892**	B.1.13	Australia	Australia	2020.21
**EPI_ISL_419813**	B.3	Australia	Australia	2020.2
**EPI_ISL_426850**	B.3	Australia	Australia	2020.22
**EPI_ISL_427160**	B.1	Australia	Australia	2020.25
**EPI_ISL_419733**	B	Australia	Australia	2020.08
**EPI_ISL_426674**	B.1.1	Australia	Australia	2020.22
**EPI_ISL_419877**	B	Australia	Australia	2020.21
**EPI_ISL_419771**	B.6.6	Australia	Australia	2020.2

**EPI_ISL_419969**	B.6	Australia	Australia	2020.22
**EPI_ISL_419967**	B.1	Australia	Australia	2020.22
**EPI_ISL_419788**	A	Australia	Australia	2020.2
**EPI_ISL_419799**	B	Australia	Australia	2020.2
**EPI_ISL_426960**	B.1.1.8	Australia	Australia	2020.23
**EPI_ISL_436689**	B.6	Australia	Australia	2020.22
**EPI_ISL_419845**	B.1	Australia	Australia	2020.21
**EPI_ISL_426844**	B.1	Australia	Australia	2020.23
**EPI_ISL_419907**	B.1.1	Australia	Australia	2020.22
**EPI_ISL_426679**	B.1.1	Australia	Australia	2020.22
**EPI_ISL_419760**	A.1	Australia	Australia	2020.19
**EPI_ISL_419938**	B.1	Australia	Australia	2020.22
**EPI_ISL_426819**	B.1	Australia	Australia	2020.23
**EPI_ISL_426775**	B.1	Australia	Australia	2020.22
**EPI_ISL_430579**	B.1	Australia	Australia	2020.26
**EPI_ISL_426914**	B.1.1.1	Australia	Australia	2020.24
**EPI_ISL_419949**	B.1	Australia	Australia	2020.22
**EPI_ISL_450214**	B.6	Australia	Australia	2020.21
**EPI_ISL_430547**	B.1	Australia	Australia	2020.25
**EPI_ISL_430569**	B.1.1	Australia	Australia	2020.27
**EPI_ISL_419987**	B.1.1.8	Australia	Australia	2020.22
**EPI_ISL_430481**	B.1	Australia	Australia	2020.25
**EPI_ISL_419755**	A	Australia	Australia	2020.19
**EPI_ISL_419722**	B.39	Australia	Australia	2020.21
**EPI_ISL_426877**	A.1	Australia	Australia	2020.24
**EPI_ISL_426954**	B.1.1	Australia	Australia	2020.24
**EPI_ISL_419806**	B.1	Australia	Australia	2020.2
**EPI_ISL_427131**	B.1.1.33	Australia	Australia	2020.26
**EPI_ISL_419816**	A.1	Australia	Australia	2020.19
**EPI_ISL_430694**	B.1.1	Australia	Australia	2020.27
**EPI_ISL_419974**	B.1	Australia	Australia	2020.22
**EPI_ISL_419948**	B.1	Australia	Australia	2020.22
**EPI_ISL_420006**	B.1.1	Australia	Australia	2020.23
**EPI_ISL_430653**	B.31	Australia	Australia	2020.23
**EPI_ISL_419887**	B.1.1.416	Australia	Australia	2020.21
**EPI_ISL_419986**	A.2.2	Australia	Australia	2020.22
**EPI_ISL_426654**	B.1	Australia	Australia	2020.22
**EPI_ISL_426646**	B.1	Australia	Australia	2020.22
**EPI_ISL_419727**	A	Australia	Australia	2020.21
**EPI_ISL_419762**	B.1	Australia	Australia	2020.19
**EPI_ISL_426661**	B.1.1	Australia	Australia	2020.22
**EPI_ISL_419814**	A	Australia	Australia	2020.21
**EPI_ISL_426747**	B.1	Australia	Australia	2020.23

**EPI_ISL_427081**	B.1.13	Australia	Australia	2020.23
**EPI_ISL_419915**	B.1	Australia	Australia	2020.22
**EPI_ISL_450212**	A	Australia	Australia	2020.21
**EPI_ISL_427145**	B.1	Australia	Australia	2020.26
**EPI_ISL_419972**	B.1	Australia	Australia	2020.22
**EPI_ISL_419886**	B.1.1	Australia	Australia	2020.21
**EPI_ISL_426688**	A.2.2	Australia	Australia	2020.22
**EPI_ISL_426934**	B.1.1	Australia	Australia	2020.24
**EPI_ISL_430639**	B.1	Australia	Australia	2020.23
**EPI_ISL_419884**	B.6	Australia	Australia	2020.21
**EPI_ISL_426648**	B.1.1.71	Australia	Australia	2020.22
**EPI_ISL_427106**	B.4	Australia	Australia	2020.26
**EPI_ISL_427046**	A.2.2	Australia	Australia	2020.22
**EPI_ISL_419855**	B.1	Australia	Australia	2020.21
**EPI_ISL_430536**	B.1	Australia	Australia	2020.25
**EPI_ISL_416514**	A.1	Australia	Australia	2020.2
**EPI_ISL_426799**	B.1	Australia	Australia	2020.23
**EPI_ISL_426955**	B.3	Australia	Australia	2020.23
**EPI_ISL_426657**	B.1	Australia	Australia	2020.22
**EPI_ISL_419859**	A.1	Australia	Australia	2020.21
**EPI_ISL_426817**	B.40	Australia	Australia	2020.23
**EPI_ISL_430610**	B.1	Australia	Australia	2020.27
**EPI_ISL_419837**	A.1	Australia	Australia	2020.2
**EPI_ISL_419968**	B.1	Australia	Australia	2020.22
**EPI_ISL_416411**	B	Australia	Australia	2020.07
**EPI_ISL_427092**	B.1.319	Australia	Australia	2020.25
**EPI_ISL_419782**	A.1	Australia	Australia	2020.2
**EPI_ISL_426793**	B.1	Australia	Australia	2020.23
**EPI_ISL_419714**	A	Australia	Australia	2020.21
**EPI_ISL_419928**	B.35	Australia	Australia	2020.22
**EPI_ISL_419821**	A	Australia	Australia	2020.21
**EPI_ISL_419748**	B.1.1.71	Australia	Australia	2020.19
**EPI_ISL_419757**	B.1	Australia	Australia	2020.19
**EPI_ISL_427016**	B.1.23	Australia	Australia	2020.24
**EPI_ISL_419781**	A.1	Australia	Australia	2020.2
**EPI_ISL_419823**	A.1	Australia	Australia	2020.22
**EPI_ISL_426656**	B.1	Australia	Australia	2020.22

Supplementary Table 2. Individual countries included in each geographical deme.

Deme	*Individual countries included in deme*
Asia	Afghanistan
	Armenia
	Azerbaijan
	Bahrain
	Bangladesh
	Bhutan
	Brunei
	Cambodia
	Georgia
	Hong Kong
	India
	Indonesia
	Iran
	Iraq
	Israel
	Japan
	Jordan
	Kazakhstan
	Kuwait
	Kyrgyzstan
	Laos
	Lebanon
	Macao
	Malaysia
	Maldives
	Mongolia
	Myanmar
	Nepal
	North Korea
	Oman
	Pakistan
	Palestine
	Philippines
	Qatar
	Saudi Arabia
	Singapore
	South Korea
	Sri Lanka
	Syria
	Taiwan
	Tajikistan
	Thailand
	Timor
	Turkey
	United Arab Emirates
	Uzbekistan
	Vietnam
	Yemen
Africa	Algeria
	Angola
	Benin
	Botswana
	Burkina Faso
	Burundi
	Cameroon
	Cape Verde
	Central African Republic
	Chad
	Comoros
	Congo
	Cote d'Ivoire
	Democratic Republic of Congo
	Djibouti
	Egypt
	Equatorial Guinea
	Eritrea
	Eswatini
	Ethiopia
	Gabon
	Gambia
	Ghana

	Guinea
	Guinea-Bissau
	Kenya
	Lesotho
	Liberia
	Libya
	Madagascar
	Malawi
	Mali
	Mauritania
	Mauritius
	Morocco
	Mozambique
	Namibia
	Niger
	Nigeria
	Rwanda
	Sao Tome and Principe
	Senegal
	Seychelles
	Sierra Leone
	Somalia
	South Africa
	South Sudan
	Sudan
	Tanzania
	Togo
	Tunisia
	Uganda
	Zambia
	Zimbabwe
Europe	Albania
	Andorra
	Austria
	Belarus
	Belgium
	Bosnia and Herzegovina
	Bulgaria
	Croatia
	Cyprus
	Czechia
	Denmark
	Estonia
	Faeroe Islands
	Finland
	France
	Germany
	Gibraltar
	Greece
	Hungary
	Iceland
	Ireland
	Isle of Man
	Italy
	Kosovo
	Latvia
	Liechtenstein
	Lithuania
	Luxembourg
	Malta
	Moldova
	Monaco
	Montenegro
	Netherlands
	North Macedonia
	Norway
	Poland
	Portugal
	Romania
	Russia
	San Marino
	Serbia
	Slovakia
	Slovenia
	Spain

	Sweden
	Switzerland
	Ukraine
	Vatican
South America	Argentina
	Bolivia
	Brazil
	Chile
	Colombia
	Ecuador
	Falkland Islands
	Guyana
	Paraguay
	Peru
	Suriname
	Uruguay
	Venezuela
North America	Anguilla
	Antigua and Barbuda
	Aruba
	Bahamas
	Barbados
	Belize
	Bermuda
	Bonaire Sint Eustatius and Saba
	British Virgin Islands
	Canada
	Cayman Islands
	Costa Rica
	Cuba
	Curacao
	Dominica
	Dominican Republic
	El Salvador
	Greenland
	Grenada
	Guatemala
	Haiti
	Honduras
	Jamaica
	Mexico
	Montserrat
	Nicaragua
	Panama
	Puerto Rico
	Saint Kitts and Nevis
	Saint Lucia
	Saint Pierre and Miquelon
	Saint Vincent and the Grenadines
	Trinidad and Tobago
	Turks and Caicos Islands
	United States
	United States Virgin Islands
Oceania	Papua New Guinea
	Northern Mariana Islands
	New Caledonia
	Marshall Islands
	Guam
	French Polynesia
	Fiji

Supplementary Table 3. Individual accession numbers, originating laboratory and submitting laboratory metadata for GISAID sequences used in this study.

Accession	Sequence originating laboratory	Sequence submitting laboratory
**EPI_ISL_460470**	Massachusetts General Hospital	Infectious Disease Program, Broad Institute of Harvard and MIT
**EPI_ISL_460433**	Massachusetts General Hospital	Infectious Disease Program, Broad Institute of Harvard and MIT
**EPI_ISL_460373**	Massachusetts General Hospital	Infectious Disease Program, Broad Institute of Harvard and MIT
**EPI_ISL_460265**	Massachusetts General Hospital	Infectious Disease Program, Broad Institute of Harvard and MIT
**EPI_ISL_460129**	Massachusetts General Hospital	Infectious Disease Program, Broad Institute of Harvard and MIT
**EPI_ISL_460126**	Massachusetts General Hospital	Infectious Disease Program, Broad Institute of Harvard and MIT
**EPI_ISL_460110**	Massachusetts General Hospital	Infectious Disease Program, Broad Institute of Harvard and MIT
**EPI_ISL_455469**	Laboratory for Respiratory Viruses, Cantacuzino National Military-Medical Institute for Research and Development	Cantacuzino Institute
**EPI_ISL_455448**	1. ViroGenetics - BSL3 Laboratory of Virology, Małopolska Centre of Biotechnology, Jagiellonian University; 2. II Department of Internal Medicine, Faculty of Medicine, Jagiellonian University Medical College; 3. Narodowy Instytut Zdrowia Publicznego - Państwowy Zakład Higieny (NIZP-PZH)	1. ViroGenetics - BSL3 Laboratory of Virology, Małopolska Centre of Biotechnology, Jagiellonian University; 2. II Department of Internal Medicine, Faculty of Medicine, Jagiellonian University Medical College; 3. Narodowy Instytut Zdrowia Publicznego - Państwowy Zakład Higieny (NIZP-PZH).
**EPI_ISL_455314**	Hospital Virgen del Rocio	Instituto de Salud Carlos III
**EPI_ISL_455123**	Dutch COVID-19 response team	Erasmus Medical Center
**EPI_ISL_454921**	Wuhan Chain Medical Labs (CMLabs)	State Key Laboratory of Biotherapy of Sichuan University
**EPI_ISL_454440**	Halmstad klinisk mikrobiologi	The Public Health Agency of Sweden
**EPI_ISL_454133**	HSE Ilha Terceira - Angra do Heroismo	Instituto Nacional de Saude (INSA)
**EPI_ISL_454089**	CH Porto - H Sto Antonio	Instituto Nacional de Saude (INSA)
**EPI_ISL_453759**	Virology Department, Sheffield Teaching Hospitals NHS Foundation Trust/Department of Infection, Immunity and Cardiovascular Disease, The Medical School, University of Sheffield	COVID-19 Genomics UK (COG-UK) Consortium
**EPI_ISL_453746**	Virology Department, Sheffield Teaching Hospitals NHS Foundation Trust/Department of Infection, Immunity and Cardiovascular Disease, The Medical School, University of Sheffield	COVID-19 Genomics UK (COG-UK) Consortium
**EPI_ISL_452815**	Virginia DCLS	Virginia DCLS
**EPI_ISL_452713**	Hospital Universitario Araba. Vitoria-Gasteiz,	SeqCOVID-SPAIN consortium/IBV(CSIC)
**EPI_ISL_452571**	Servicio de Microbiolog√≠a y Parasitolog√≠a cl√≠nica. UCEIMP. Hospital Universitario Virgen del Roc√≠o/IBIS/CSIC/US.	SeqCOVID-SPAIN consortium/IBV(CSIC)
**EPI_ISL_452284**	Michigan Department of Health and Human Services, Bureau of Laboratories	Michigan Department of Health and Human Services, Bureau of Laboratories
**EPI_ISL_452154**	Utah Public Health Laboratory	Utah Public Health Laboratory
**EPI_ISL_451935**	CUB Hopital Erasme Laboratoire d'Anatomie Pathologique	CUB Hopital Erasme Laboratoire d'Anatomie Pathologique
**EPI_ISL_451835**	Viollier AG	Department of Biosystems Science and Engineering, ETH Z√ºrich
**EPI_ISL_451814**	Viollier AG	Department of Biosystems Science and Engineering, ETH Z√ºrich
**EPI_ISL_451719**	Viollier AG	Department of Biosystems Science and Engineering, ETH Z√ºrich
**EPI_ISL_451619**	Pathology West - NSW Health Pathology	NSW Health Pathology - Institute of Clinical Pathology and Medical Research
**EPI_ISL_451595**	ACT pathology	NSW Health Pathology - Institute of Clinical Pathology and Medical Research
**EPI_ISL_451306**	Molecular Virology Unit, Fondazione IRCCS Policlinico San Matteo , Pavia	Laboratory of Virology, INMI Lazzaro Spallanzani IRCCS

**EPI_ISL_451278**	LSUHS Emerging Viral Threat Laboratory	Microbial Genome Sequencing Center
**EPI_ISL_450784**	Government Medical College-Bhavnagar	Gujarat Biotechnology Research Centre
**EPI_ISL_450307**	H√¥pital du Suro√Æt	Laboratoire de sant√© publique du Qu√©bec
**EPI_ISL_450265**	WHO National Influenza Centre Russian Federation	WHO National Influenza Centre Russian Federation
**EPI_ISL_450231**	Robert Garry lab	Andersen lab at Scripps Research
**EPI_ISL_450214**	unknown	Microbiological Diagnostic Unit Public Health Laboratory (MDU-PHL) and Victorian Infectious Disease Reference Laboratory (VIDRL)
**EPI_ISL_450212**	unknown	Microbiological Diagnostic Unit Public Health Laboratory (MDU-PHL) and Victorian Infectious Disease Reference Laboratory (VIDRL)
**EPI_ISL_450177**	Robert Garry lab	Andersen lab at Scripps Research
**EPI_ISL_450010**	MSHS Clinical Microbiology Laboratories	MSHS Pathogen Surveillance Program
**EPI_ISL_449654**	University College London, Great Ormond Street Hospital for Children NHS Foundation Trust, Imperial College Healthcare NHS Trust	COVID-19 Genomics UK (COG-UK) Consortium
**EPI_ISL_449109**	Quadram Institute Bioscience	COVID-19 Genomics UK (COG-UK) Consortium
**EPI_ISL_449035**	Quadram Institute Bioscience	COVID-19 Genomics UK (COG-UK) Consortium
**EPI_ISL_449017**	Quadram Institute Bioscience	COVID-19 Genomics UK (COG-UK) Consortium
**EPI_ISL_448980**	Quadram Institute Bioscience	COVID-19 Genomics UK (COG-UK) Consortium
**EPI_ISL_448787**	Oxford Viromics, NDM, University of Oxford	
**EPI_ISL_448457**	Oxford Viromics, NDM, University of Oxford	
**EPI_ISL_448369**	Quadram Institute Bioscience	COVID-19 Genomics UK (COG-UK) Consortium
**EPI_ISL_448299**	Quadram Institute Bioscience	COVID-19 Genomics UK (COG-UK) Consortium
**EPI_ISL_447863**	CSIR-Centre for Cellular and Molecular Biology	CSIR-Centre for Cellular and Molecular Biology
**EPI_ISL_447816**	Instituto Nacional de Salud, Bogot√°, Colombia	Grupo de Investigaciones Microbiol√≥gicas-UR (GIMUR), Departamento de Biolog√≠a, Facultad de Ciencias Naturales, Universidad del Rosario, Bogot√°, Colombia Instituto Nacional de Salud, Bogot√°, Colombia Icahn School of Medicine at Mount Sinai, New York, USA
**EPI_ISL_447781**	Instituto Nacional de Salud, Bogot√°, Colombia	Grupo de Investigaciones Microbiol√≥gicas-UR (GIMUR), Departamento de Biolog√≠a, Facultad de Ciencias Naturales, Universidad del Rosario, Bogot√°, Colombia Instituto Nacional de Salud, Bogot√°, Colombia Icahn School of Medicine at Mount Sinai, New York, USA
**EPI_ISL_447589**	University of Florida, Lednicky Lab	University of Florida, Lednicky Lab
**EPI_ISL_447485**	Servicio de Microbiologia. Hospital Clinico Universitario de Valencia	Sequencing and Bioinformatics Service and Molecular Epidemiology Research Group. FISABIO-Public Health
**EPI_ISL_447450**	Clinical Microbiology Laboratory, Sheba Medical Center	Stern Lab
**EPI_ISL_447384**	Clinical Microbiology Laboratory, The Baruch Padeh Medical Center, Poriya	Stern Lab
**EPI_ISL_447324**	Clinical Virology Laboratory, Soroka Medical Center and the Faculty of Health Sciences, Ben-Gurion University of the Negev	Stern Lab
**EPI_ISL_447280**	Microbiology laboratory, Assuta Ashdod University-Affiliated Hospital	Stern Lab
**EPI_ISL_447221**	Michigan Department of Health and Human Services, Bureau of Laboratories	Michigan Department of Health and Human Services, Bureau of Laboratories
**EPI_ISL_447173**	Michigan Department of Health and Human Services, Bureau of Laboratories	Michigan Department of Health and Human Services, Bureau of Laboratories

**EPI_ISL_447098**	Michigan Department of Health and Human Services, Bureau of Laboratories	Michigan Department of Health and Human Services, Bureau of Laboratories
**EPI_ISL_447059**	Michigan Department of Health and Human Services, Bureau of Laboratories	Michigan Department of Health and Human Services, Bureau of Laboratories
**EPI_ISL_445617**	Wales Specialist Virology Centre	Public Health Wales Microbiology Cardiff
**EPI_ISL_445220**	Laborarory for Respiratory Viruses, "Cantacuzino" National Military-Medical Institute for Resararch and Development	Cantacuzino Institute
**EPI_ISL_445130**	Robert Garry lab	Andersen lab at Scripps Research
**EPI_ISL_444384**	Department of Pathology, University of Cambridge	COVID-19 Genomics UK (COG-UK) Consortium
**EPI_ISL_444285**	University of Birmingham	COVID-19 Genomics UK (COG-UK) Consortium
**EPI_ISL_444162**	University College London, Great Ormond Street Hospital for Children NHS Foundation Trust, Imperial College Healthcare NHS Trust	COVID-19 Genomics UK (COG-UK) Consortium
**EPI_ISL_444134**	University College London, Great Ormond Street Hospital for Children NHS Foundation Trust, Imperial College Healthcare NHS Trust	COVID-19 Genomics UK (COG-UK) Consortium
**EPI_ISL_443717**	PHE South West Regional Laboratory, National Infection Service	Wellcome Sanger Institute for the COVID-19 Genomics UK (COG-UK) consortium
**EPI_ISL_443545**	Department of Pathology, University of Cambridge	Wellcome Sanger Institute for the COVID-19 Genomics UK (COG-UK) consortium
**EPI_ISL_442764**	PHE South West Regional Laboratory, National Infection Service	Wellcome Sanger Institute for the COVID-19 Genomics UK (COG-UK) consortium
**EPI_ISL_442729**	PHE South West Regional Laboratory, National Infection Service	Wellcome Sanger Institute for the COVID-19 Genomics UK (COG-UK) consortium
**EPI_ISL_442706**	PHE South West Regional Laboratory, National Infection Service	Wellcome Sanger Institute for the COVID-19 Genomics UK (COG-UK) consortium
**EPI_ISL_442370**	Virology Department, Sheffield Teaching Hospitals NHS Foundation Trust/Department of Infection, Immunity and Cardiovascular Disease, The Medical School, University of Sheffield	COVID-19 Genomics UK (COG-UK) Consortium
**EPI_ISL_442338**	Department of Pathology, University of Cambridge	Wellcome Sanger Institute for the COVID-19 Genomics UK (COG-UK) consortium
**EPI_ISL_441975**	Virology Department, Sheffield Teaching Hospitals NHS Foundation Trust/Department of Infection, Immunity and Cardiovascular Disease, The Medical School, University of Sheffield	COVID-19 Genomics UK (COG-UK) Consortium
**EPI_ISL_441564**	Department of Pathology, University of Cambridge	Wellcome Sanger Institute for the COVID-19 Genomics UK (COG-UK) consortium
**EPI_ISL_441269**	Department of Pathology, University of Cambridge	Wellcome Sanger Institute for the COVID-19 Genomics UK (COG-UK) consortium
**EPI_ISL_441158**	Department of Pathology, University of Cambridge	Wellcome Sanger Institute for the COVID-19 Genomics UK (COG-UK) consortium
**EPI_ISL_441125**	Department of Pathology, University of Cambridge	Wellcome Sanger Institute for the COVID-19 Genomics UK (COG-UK) consortium
**EPI_ISL_439788**	Liverpool Clinical Laboratories	COVID-19 Genomics UK (COG-UK) Consortium
**EPI_ISL_439656**	Department of Pathology, University of Cambridge	Wellcome Sanger Institute for the COVID-19 Genomics UK (COG-UK) consortium
**EPI_ISL_439424**	Department of Pathology, University of Cambridge	Wellcome Sanger Institute for the COVID-19 Genomics UK (COG-UK) consortium
**EPI_ISL_439347**	Virology Department, Royal Infirmary of Edinburgh, NHS Lothian / School of Biological Sciences, University of Edinburgh / Institute of Genetics and Molecular Medicine, University of Edinburgh	COVID-19 Genomics UK (COG-UK) Consortium
**EPI_ISL_439261**	Virology Department, Royal Infirmary of Edinburgh, NHS Lothian / School of Biological Sciences, University of Edinburgh / Institute of Genetics and Molecular Medicine, University of Edinburgh	COVID-19 Genomics UK (COG-UK) Consortium
**EPI_ISL_439207**	Virology Department, Royal Infirmary of Edinburgh, NHS Lothian / School of Biological Sciences, University of Edinburgh / Institute of	COVID-19 Genomics UK (COG-UK) Consortium

	Genetics and Molecular Medicine, University of Edinburgh	
**EPI_ISL_439188**	Virology Department, Royal Infirmary of Edinburgh, NHS Lothian / School of Biological Sciences, University of Edinburgh / Institute of Genetics and Molecular Medicine, University of Edinburgh	COVID-19 Genomics UK (COG-UK) Consortium
**EPI_ISL_438910**	West of Scotland Specialist Virology Centre, NHSGGC / MRC-University of Glasgow Centre for Virus Research	COVID-19 Genomics UK (COG-UK) Consortium
**EPI_ISL_438701**	Department of Pathology, University of Cambridge	COVID-19 Genomics UK (COG-UK) Consortium
**EPI_ISL_438590**	Department of Pathology, University of Cambridge	COVID-19 Genomics UK (COG-UK) Consortium
**EPI_ISL_438315**	Department of Pathology, University of Cambridge	Wellcome Sanger Institute for the COVID-19 Genomics UK (COG-UK) consortium
**EPI_ISL_438309**	Department of Pathology, University of Cambridge	Wellcome Sanger Institute for the COVID-19 Genomics UK (COG-UK) consortium
**EPI_ISL_438299**	Department of Pathology, University of Cambridge	Wellcome Sanger Institute for the COVID-19 Genomics UK (COG-UK) consortium
**EPI_ISL_438225**	Johns Hopkins Hospital Department of Pathology	Johns Hopkins Hospital Department of Pathology
**EPI_ISL_438103**	Center for Virology, Medical University of Vienna	Bergthaler laboratory, CeMM Research Center for Molecular Medicine of the Austrian Academy of Sciences
**EPI_ISL_437760**	Pathogen Genomics Lab King Abdullah University of Science and Technology(KAUST)	Pathogen Genomics Lab King Abdullah University of Science and Technology(KAUST)
**EPI_ISL_437356**	Viral Respiratory Lab, National Institute for Biomedical Research (INRB)	Pathogen Sequencing Lab, National Institute for Biomedical Research (INRB)
**EPI_ISL_437203**	Diagnostic- and Research Institute of Pathology, Medical University of Graz	Diagnostic- and Research Institute of Pathology, Medical University of Graz
**EPI_ISL_437093**	Riga East Clinical University Hospital	Latvian Biomedical Research and Study Centre
**EPI_ISL_437031**	Department of Virus and Microbiological Special Diagnostics, Statens Serum Institut, Copenhagen, Denmark, Artillerivej 5, 2300 Copenahgen S	Albertsen lab, Department of Chemistry and Bioscience, Aalborg University, Denmark
**EPI_ISL_437001**	Department of Virus and Microbiological Special Diagnostics, Statens Serum Institut, Copenhagen, Denmark, Artillerivej 5, 2300 Copenahgen S	Albertsen lab, Department of Chemistry and Bioscience, Aalborg University, Denmark
**EPI_ISL_436717**	Genomics and Computational Biology Lab, Scientific Research Institute of Physical-Chemical Medicine, FMBA of Russia	Genomics and Computational Biology Lab, Scientific Research Institute of Physical-Chemical Medicine, FMBA of Russia
**EPI_ISL_436715**	Genomics and Computational Biology Lab, Scientific Research Institute of Physical-Chemical Medicine, FMBA of Russia	Genomics and Computational Biology Lab, Scientific Research Institute of Physical-Chemical Medicine, FMBA of Russia
**EPI_ISL_436689**	Victorian Infectious Diseases Reference Laboratory (VIDRL)	Microbiological Diagnostic Unit Public Health Laboratory and Victorian Infectious Diseases Reference Laboratory, The Peter Doherty Institute for Infection & Immunity
**EPI_ISL_436656**	County of Santa Clara Public Health Department	Chan-Zuckerberg Biohub
**EPI_ISL_436631**	University of Wisconsin-Madison AIDS Vaccine Research Laboratories	University of Wisconsin-Madison AIDS Vaccine Research Laboratories
**EPI_ISL_436485**	UPMC Clinical Laboratory	Microbial Genome Sequencing Center, Microbial Genomic Epidemiological Laboratory
**EPI_ISL_435540**	NYU Langone Health	Departments of Pathology and Medicine, New York University School of Medicine
**EPI_ISL_435006**	Houston Methodist Hospital	Houston Methodist Hospital
**EPI_ISL_435000**	Houston Methodist Hospital	Houston Methodist Hospital
**EPI_ISL_434908**	Houston Methodist Hospital	Houston Methodist Hospital
**EPI_ISL_434516**	Biolab Diagnostic Laboratories	Andersen lab at Scripps Research
**EPI_ISL_434320**	Washington State Department of Health	Seattle Flu Study
**EPI_ISL_434136**	Washington State Department of Health	Seattle Flu Study
**EPI_ISL_433928**	Department of Pathology, University of Cambridge	COVID-19 Genomics UK (COG-UK) Consortium

**EPI_ISL_433829**	Department of Pathology, University of Cambridge	COVID-19 Genomics UK (COG-UK) Consortium
**EPI_ISL_433828**	Department of Pathology, University of Cambridge	COVID-19 Genomics UK (COG-UK) Consortium
**EPI_ISL_433595**	West of Scotland Specialist Virology Centre, NHSGGC / MRC-University of Glasgow Centre for Virus Research	COVID-19 Genomics UK (COG-UK) Consortium
**EPI_ISL_433374**	Virology Department, Royal Infirmary of Edinburgh, NHS Lothian / School of Biological Sciences, University of Edinburgh / Institute of Genetics and Molecular Medicine, University of Edinburgh	COVID-19 Genomics UK (COG-UK) Consortium
**EPI_ISL_432642**	Virology Department, Sheffield Teaching Hospitals NHS Foundation Trust / Virology Department, Sheffield Teaching Hospitals NHS Foundation Trust	COVID-19 Genomics UK (COG-UK) Consortium
**EPI_ISL_432622**	Virology Department, Sheffield Teaching Hospitals NHS Foundation Trust / Virology Department, Sheffield Teaching Hospitals NHS Foundation Trust	COVID-19 Genomics UK (COG-UK) Consortium
**EPI_ISL_432390**	Wales Specialist Virology Centre	Public Health Wales Microbiology Cardiff
**EPI_ISL_430719**	Hospital Universitario La Paz	Hospital Universitario 12 de Octubre
**EPI_ISL_430694**	Victorian Infectious Diseases Reference Laboratory (VIDRL)	Microbiological Diagnostic Unit Public Health Laboratory and Victorian Infectious Diseases Reference Laboratory, The Peter Doherty Institute for Infection and Immunity
**EPI_ISL_430653**	Microbiological Diagnostic Unit Public Health Laboratory	Microbiological Diagnostic Unit Public Health Laboratory
**EPI_ISL_430639**	Microbiological Diagnostic Unit Public Health Laboratory	Microbiological Diagnostic Unit Public Health Laboratory
**EPI_ISL_430610**	Victorian Infectious Diseases Reference Laboratory (VIDRL)	Microbiological Diagnostic Unit Public Health Laboratory and Victorian Infectious Diseases Reference Laboratory, The Peter Doherty Institute for Infection and Immunity
**EPI_ISL_430579**	Victorian Infectious Diseases Reference Laboratory (VIDRL)	Microbiological Diagnostic Unit Public Health Laboratory and Victorian Infectious Diseases Reference Laboratory, The Peter Doherty Institute for Infection and Immunity
**EPI_ISL_430569**	Victorian Infectious Diseases Reference Laboratory (VIDRL)	Microbiological Diagnostic Unit Public Health Laboratory and Victorian Infectious Diseases Reference Laboratory, The Peter Doherty Institute for Infection and Immunity
**EPI_ISL_430547**	Victorian Infectious Diseases Reference Laboratory (VIDRL)	Microbiological Diagnostic Unit Public Health Laboratory and Victorian Infectious Diseases Reference Laboratory, The Peter Doherty Institute for Infection and Immunity
**EPI_ISL_430545**	Victorian Infectious Diseases Reference Laboratory (VIDRL)	Microbiological Diagnostic Unit Public Health Laboratory and Victorian Infectious Diseases Reference Laboratory, The Peter Doherty Institute for Infection and Immunity
**EPI_ISL_430536**	Victorian Infectious Diseases Reference Laboratory (VIDRL)	Microbiological Diagnostic Unit Public Health Laboratory and Victorian Infectious Diseases Reference Laboratory, The Peter Doherty Institute for Infection and Immunity
**EPI_ISL_430481**	Victorian Infectious Diseases Reference Laboratory (VIDRL)	Microbiological Diagnostic Unit Public Health Laboratory and Victorian Infectious Diseases Reference Laboratory, The Peter Doherty Institute for Infection and Immunity
**EPI_ISL_430430**	NYU Langone Health	Departments of Pathology and Medicine, New York University School of Medicine
**EPI_ISL_430415**	NYU Langone Health	Departments of Pathology and Medicine, New York University School of Medicine
**EPI_ISL_430353**	NYU Langone Health	Departments of Pathology and Medicine, New York University School of Medicine
**EPI_ISL_430340**	NYU Langone Health	Departments of Pathology and Medicine, New York University School of Medicine

**EPI_ISL_430261**	Washington State Department of Health	Seattle Flu Study
**EPI_ISL_430245**	Washington State Department of Health	Seattle Flu Study
**EPI_ISL_429464**	Department of Virus and Microbiological Special Diagnostics, Statens Serum Institut, Copenhagen, Denmark, Artillerivej 5, 2300 Copenahgen S	Albertsen lab, Department of Chemistry and Bioscience, Aalborg University, Denmark
**EPI_ISL_429431**	Department of Virus and Microbiological Special Diagnostics, Statens Serum Institut, Copenhagen, Denmark, Artillerivej 5, 2300 Copenahgen S	Albertsen lab, Department of Chemistry and Bioscience, Aalborg University, Denmark
**EPI_ISL_429277**	Department of Clinical Microbiology, Copenhagen University Hospital, Hvidovre, Kettegaard Alle 30, 2650 Hvidovre.	Albertsen lab, Department of Chemistry and Bioscience, Aalborg University, Denmark
**EPI_ISL_428921**	State Research Center of Virology and Biotechnology VECTOR, Department of Collection of Microorganisms	State Research Center of Virology and Biotechnology VECTOR, Department of Collection of Microorganisms
**EPI_ISL_428862**	State Research Center of Virology and Biotechnology VECTOR, Department of Collection of Microorganisms	State Research Center of Virology and Biotechnology VECTOR, Department of Collection of Microorganisms
**EPI_ISL_428348**	Maison de Sant√© du Val d'Ormois	National Reference Center for Viruses of Respiratory Infections, Institut Pasteur, Paris
**EPI_ISL_428301**	University of Wisconsin-Madison AIDS Vaccine Research Laboratories	University of Wisconsin-Madison AIDS Vaccine Research Laboratories
**EPI_ISL_427773**	Centre for Infectious Diseases and Microbiology Public Health	NSW Health Pathology - Institute of Clinical Pathology and Medical Research
**EPI_ISL_427746**	Centre for Infectious Diseases and Microbiology Public Health	NSW Health Pathology - Institute of Clinical Pathology and Medical Research
**EPI_ISL_427607**	NewYork-Presbyterian & Mason Lab	Mason Lab
**EPI_ISL_427485**	NYU Langone Health	Departments of Pathology and Medicine, New York University School of Medicine
**EPI_ISL_427160**	Victorian Infectious Diseases Reference Laboratory (VIDRL)	Microbiological Diagnostic Unit Public Health Laboratory and Victorian Infectious Diseases Reference Laboratory, Doherty Institute
**EPI_ISL_427145**	Victorian Infectious Diseases Reference Laboratory (VIDRL)	Microbiological Diagnostic Unit Public Health Laboratory and Victorian Infectious Diseases Reference Laboratory, Doherty Institute
**EPI_ISL_427131**	Victorian Infectious Diseases Reference Laboratory (VIDRL)	Microbiological Diagnostic Unit Public Health Laboratory and Victorian Infectious Diseases Reference Laboratory, Doherty Institute
**EPI_ISL_427106**	Victorian Infectious Diseases Reference Laboratory (VIDRL)	Microbiological Diagnostic Unit Public Health Laboratory and Victorian Infectious Diseases Reference Laboratory, Doherty Institute
**EPI_ISL_427095**	Victorian Infectious Diseases Reference Laboratory (VIDRL)	Microbiological Diagnostic Unit Public Health Laboratory and Victorian Infectious Diseases Reference Laboratory, Doherty Institute
**EPI_ISL_427092**	Victorian Infectious Diseases Reference Laboratory (VIDRL)	Microbiological Diagnostic Unit Public Health Laboratory and Victorian Infectious Diseases Reference Laboratory, Doherty Institute
**EPI_ISL_427081**	Victorian Infectious Diseases Reference Laboratory (VIDRL)	Microbiological Diagnostic Unit Public Health Laboratory and Victorian Infectious Diseases Reference Laboratory, Doherty Institute
**EPI_ISL_427046**	Victorian Infectious Diseases Reference Laboratory (VIDRL)	Microbiological Diagnostic Unit Public Health Laboratory and Victorian Infectious Diseases Reference Laboratory, Doherty Institute
**EPI_ISL_427025**	Victorian Infectious Diseases Reference Laboratory (VIDRL)	Microbiological Diagnostic Unit Public Health Laboratory and Victorian Infectious Diseases Reference Laboratory, Doherty Institute
**EPI_ISL_427016**	Victorian Infectious Diseases Reference Laboratory (VIDRL)	Microbiological Diagnostic Unit Public Health Laboratory and Victorian Infectious Diseases Reference Laboratory, Doherty Institute
**EPI_ISL_426977**	Victorian Infectious Diseases Reference Laboratory (VIDRL)	Microbiological Diagnostic Unit Public Health Laboratory and Victorian Infectious Diseases Reference Laboratory, Doherty Institute
**EPI_ISL_426971**	Victorian Infectious Diseases Reference Laboratory (VIDRL)	Microbiological Diagnostic Unit Public Health Laboratory and Victorian Infectious Diseases Reference Laboratory, Doherty Institute

**EPI_ISL_426960**	Victorian Infectious Diseases Reference Laboratory (VIDRL)	Microbiological Diagnostic Unit Public Health Laboratory and Victorian Infectious Diseases Reference Laboratory, Doherty Institute
**EPI_ISL_426955**	Victorian Infectious Diseases Reference Laboratory (VIDRL)	Microbiological Diagnostic Unit Public Health Laboratory and Victorian Infectious Diseases Reference Laboratory, Doherty Institute
**EPI_ISL_426954**	Victorian Infectious Diseases Reference Laboratory (VIDRL)	Microbiological Diagnostic Unit Public Health Laboratory and Victorian Infectious Diseases Reference Laboratory, Doherty Institute
**EPI_ISL_426934**	Victorian Infectious Diseases Reference Laboratory (VIDRL)	Microbiological Diagnostic Unit Public Health Laboratory and Victorian Infectious Diseases Reference Laboratory, Doherty Institute
**EPI_ISL_426914**	Microbiological Diagnostic Unit Public Health Laboratory	Microbiological Diagnostic Unit Public Health Laboratory
**EPI_ISL_426890**	Motol University Hospital	Institute of Applied Biotechnologies a.s.
**EPI_ISL_426877**	Victorian Infectious Diseases Reference Laboratory (VIDRL)	Microbiological Diagnostic Unit Public Health Laboratory and Victorian Infectious Diseases Reference Laboratory, Doherty Institute
**EPI_ISL_426850**	Victorian Infectious Diseases Reference Laboratory (VIDRL)	Microbiological Diagnostic Unit Public Health Laboratory and Victorian Infectious Diseases Reference Laboratory, Doherty Institute
**EPI_ISL_426844**	Victorian Infectious Diseases Reference Laboratory (VIDRL)	Microbiological Diagnostic Unit Public Health Laboratory and Victorian Infectious Diseases Reference Laboratory, Doherty Institute
**EPI_ISL_426819**	Victorian Infectious Diseases Reference Laboratory (VIDRL)	Microbiological Diagnostic Unit Public Health Laboratory and Victorian Infectious Diseases Reference Laboratory, Doherty Institute
**EPI_ISL_426817**	Victorian Infectious Diseases Reference Laboratory (VIDRL)	Microbiological Diagnostic Unit Public Health Laboratory and Victorian Infectious Diseases Reference Laboratory, Doherty Institute
**EPI_ISL_426799**	Victorian Infectious Diseases Reference Laboratory (VIDRL)	Microbiological Diagnostic Unit Public Health Laboratory and Victorian Infectious Diseases Reference Laboratory, Doherty Institute
**EPI_ISL_426793**	Victorian Infectious Diseases Reference Laboratory (VIDRL)	Microbiological Diagnostic Unit Public Health Laboratory and Victorian Infectious Diseases Reference Laboratory, Doherty Institute
**EPI_ISL_426782**	Victorian Infectious Diseases Reference Laboratory (VIDRL)	Microbiological Diagnostic Unit Public Health Laboratory and Victorian Infectious Diseases Reference Laboratory, Doherty Institute
**EPI_ISL_426781**	Victorian Infectious Diseases Reference Laboratory (VIDRL)	Microbiological Diagnostic Unit Public Health Laboratory and Victorian Infectious Diseases Reference Laboratory, Doherty Institute
**EPI_ISL_426775**	Victorian Infectious Diseases Reference Laboratory (VIDRL)	Microbiological Diagnostic Unit Public Health Laboratory and Victorian Infectious Diseases Reference Laboratory, Doherty Institute
**EPI_ISL_426747**	Victorian Infectious Diseases Reference Laboratory (VIDRL)	Microbiological Diagnostic Unit Public Health Laboratory and Victorian Infectious Diseases Reference Laboratory, Doherty Institute
**EPI_ISL_426693**	Victorian Infectious Diseases Reference Laboratory (VIDRL)	Microbiological Diagnostic Unit Public Health Laboratory and Victorian Infectious Diseases Reference Laboratory, Doherty Institute
**EPI_ISL_426688**	Victorian Infectious Diseases Reference Laboratory (VIDRL)	Microbiological Diagnostic Unit Public Health Laboratory and Victorian Infectious Diseases Reference Laboratory, Doherty Institute
**EPI_ISL_426683**	Victorian Infectious Diseases Reference Laboratory (VIDRL)	Microbiological Diagnostic Unit Public Health Laboratory and Victorian Infectious Diseases Reference Laboratory, Doherty Institute
**EPI_ISL_426679**	Victorian Infectious Diseases Reference Laboratory (VIDRL)	Microbiological Diagnostic Unit Public Health Laboratory and Victorian Infectious Diseases Reference Laboratory, Doherty Institute
**EPI_ISL_426674**	Victorian Infectious Diseases Reference Laboratory (VIDRL)	Microbiological Diagnostic Unit Public Health Laboratory and Victorian Infectious Diseases Reference Laboratory, Doherty Institute
**EPI_ISL_426661**	Victorian Infectious Diseases Reference Laboratory (VIDRL)	Microbiological Diagnostic Unit Public Health Laboratory and Victorian Infectious Diseases Reference Laboratory, Doherty Institute

**EPI_ISL_426657**	Victorian Infectious Diseases Reference Laboratory (VIDRL)	Microbiological Diagnostic Unit Public Health Laboratory and Victorian Infectious Diseases Reference Laboratory, Doherty Institute
**EPI_ISL_426656**	Victorian Infectious Diseases Reference Laboratory (VIDRL)	Microbiological Diagnostic Unit Public Health Laboratory and Victorian Infectious Diseases Reference Laboratory, Doherty Institute
**EPI_ISL_426654**	Victorian Infectious Diseases Reference Laboratory (VIDRL)	Microbiological Diagnostic Unit Public Health Laboratory and Victorian Infectious Diseases Reference Laboratory, Doherty Institute
**EPI_ISL_426648**	Victorian Infectious Diseases Reference Laboratory (VIDRL)	Microbiological Diagnostic Unit Public Health Laboratory and Victorian Infectious Diseases Reference Laboratory, Doherty Institute
**EPI_ISL_426646**	Victorian Infectious Diseases Reference Laboratory (VIDRL)	Microbiological Diagnostic Unit Public Health Laboratory and Victorian Infectious Diseases Reference Laboratory, Doherty Institute
**EPI_ISL_426530**	AZ SPHL, Arizona Department of Health Services	TGen North
**EPI_ISL_426100**	UW Virology Lab	UW Virology Lab
**EPI_ISL_426059**	Center of Medical Microbiology, Virology, and Hospital Hygiene, University of Duesseldorf	Center of Medical Microbiology, Virology, and Hospital Hygiene, University of Duesseldorf
**EPI_ISL_425875**	Virology Department, Royal Infirmary of Edinburgh, NHS Lothian / School of Biological Sciences, University of Edinburgh / Institute of Genetics and Molecular Medicine, University of Edinburgh	COVID-19 Genomics UK (COG-UK) Consortium
**EPI_ISL_425724**	West of Scotland Specialist Virology Centre, NHSGGC / MRC-University of Glasgow Centre for Virus Research	COVID-19 Genomics UK (COG-UK) Consortium
**EPI_ISL_425183**	Servicio de Microbiolog√≠a. Consorcio Hospital General Universitario de Valencia	Sequencing and Bioinformatics Service and Molecular Epidemiology Research Group. FISABIO-Public Health
**EPI_ISL_424901**	NJ Public Health and Environmental Laboratories	Pathogen Discovery, Respiratory Viruses Branch, Division of Viral Diseases, Centers for Disease Control and Prevention
**EPI_ISL_424435**	The National University Hospital of Iceland	deCODE genetics
**EPI_ISL_424356**	Beijing Institute of Microbiology and Epidemiology	Beijing Institute of Microbiology and Epidemiology
**EPI_ISL_424221**	UW Virology Lab	UW Virology Lab
**EPI_ISL_423938**	Respiratory Virus Unit, Microbiology Services Colindale, Public Health England	Respiratory Virus Unit, Microbiology Services Colindale, Public Health England
**EPI_ISL_423481**	Respiratory Virus Unit, Microbiology Services Colindale, Public Health England	Respiratory Virus Unit, Microbiology Services Colindale, Public Health England
**EPI_ISL_423435**	Respiratory Virus Unit, Microbiology Services Colindale, Public Health England	Respiratory Virus Unit, Microbiology Services Colindale, Public Health England
**EPI_ISL_423244**	Respiratory Virus Unit, Microbiology Services Colindale, Public Health England	Respiratory Virus Unit, Microbiology Services Colindale, Public Health England
**EPI_ISL_423144**	Respiratory Virus Unit, Microbiology Services Colindale, Public Health England	Respiratory Virus Unit, Microbiology Services Colindale, Public Health England
**EPI_ISL_422919**	Dutch COVID-19 response team	Erasmus Medical Center
**EPI_ISL_422684**	Dutch COVID-19 response team	Erasmus Medical Center
**EPI_ISL_422515**	MSHS Clinical Microbiology Laboratories	MSHS Pathogen Surveillance Program
**EPI_ISL_422178**	Wales Specialist Virology Centre	Public Health Wales Microbiology Cardiff
**EPI_ISL_422053**	Wales Specialist Virology Centre	Public Health Wales Microbiology Cardiff
**EPI_ISL_421879**	Respiratory Virus Unit, Microbiology Services Colindale, Public Health England	Respiratory Virus Unit, Microbiology Services Colindale, Public Health England
**EPI_ISL_421712**	NYU Langone Health	Departments of Pathology and Medicine, New York University School of Medicine
**EPI_ISL_421633**	MSHS Clinical Microbiology Laboratories	MSHS Pathogen Surveillance Program
**EPI_ISL_421623**	MSHS Clinical Microbiology Laboratories	MSHS Pathogen Surveillance Program
**EPI_ISL_421497**	INSA	Instituto Nacional de Saude (INSA)
**EPI_ISL_421457**	H Dr Nelio Mendonca - Funchal	Instituto Nacional de Saude (INSA)
**EPI_ISL_421243**	Jiangxi Province Center for Disease Control and Prevention	Jiangxi Province Center for Disease Control and Prevention

**EPI_ISL_420240**	Virology Department, Sheffield Teaching Hospitals NHS Foundation Trust	Department of Infection, Immunity and Cardiovascular Disease, The Florey Institute, The Medical School, University of Sheffield
**EPI_ISL_420019**	Virginia DCLS	Virginia DCLS
**EPI_ISL_420011**	Microbiological Diagnostic Unit Public Health Laboratory	Microbiological Diagnostic Unit Public Health Laboratory
**EPI_ISL_420006**	Microbiological Diagnostic Unit Public Health Laboratory	Microbiological Diagnostic Unit Public Health Laboratory
**EPI_ISL_419987**	Victorian Infectious Diseases Reference Laboratory (VIDRL)	Victorian Infectious Diseases Reference Laboratory and Microbiological Diagnostic Unit Public Health Laboratory, Doherty Institute
**EPI_ISL_419986**	Victorian Infectious Diseases Reference Laboratory (VIDRL)	Victorian Infectious Diseases Reference Laboratory and Microbiological Diagnostic Unit Public Health Laboratory, Doherty Institute
**EPI_ISL_419974**	Victorian Infectious Diseases Reference Laboratory (VIDRL)	Victorian Infectious Diseases Reference Laboratory and Microbiological Diagnostic Unit Public Health Laboratory, Doherty Institute
**EPI_ISL_419972**	Victorian Infectious Diseases Reference Laboratory (VIDRL)	Victorian Infectious Diseases Reference Laboratory and Microbiological Diagnostic Unit Public Health Laboratory, Doherty Institute
**EPI_ISL_419969**	Victorian Infectious Diseases Reference Laboratory (VIDRL)	Victorian Infectious Diseases Reference Laboratory and Microbiological Diagnostic Unit Public Health Laboratory, Doherty Institute
**EPI_ISL_419968**	Victorian Infectious Diseases Reference Laboratory (VIDRL)	Victorian Infectious Diseases Reference Laboratory and Microbiological Diagnostic Unit Public Health Laboratory, Doherty Institute
**EPI_ISL_419967**	Victorian Infectious Diseases Reference Laboratory (VIDRL)	Victorian Infectious Diseases Reference Laboratory and Microbiological Diagnostic Unit Public Health Laboratory, Doherty Institute
**EPI_ISL_419949**	Victorian Infectious Diseases Reference Laboratory (VIDRL)	Victorian Infectious Diseases Reference Laboratory and Microbiological Diagnostic Unit Public Health Laboratory, Doherty Institute
**EPI_ISL_419948**	Victorian Infectious Diseases Reference Laboratory (VIDRL)	Victorian Infectious Diseases Reference Laboratory and Microbiological Diagnostic Unit Public Health Laboratory, Doherty Institute
**EPI_ISL_419938**	Victorian Infectious Diseases Reference Laboratory (VIDRL)	Victorian Infectious Diseases Reference Laboratory and Microbiological Diagnostic Unit Public Health Laboratory, Doherty Institute
**EPI_ISL_419937**	Victorian Infectious Diseases Reference Laboratory (VIDRL)	Victorian Infectious Diseases Reference Laboratory and Microbiological Diagnostic Unit Public Health Laboratory, Doherty Institute
**EPI_ISL_419935**	Victorian Infectious Diseases Reference Laboratory (VIDRL)	Victorian Infectious Diseases Reference Laboratory and Microbiological Diagnostic Unit Public Health Laboratory, Doherty Institute
**EPI_ISL_419928**	Victorian Infectious Diseases Reference Laboratory (VIDRL)	Victorian Infectious Diseases Reference Laboratory and Microbiological Diagnostic Unit Public Health Laboratory, Doherty Institute
**EPI_ISL_419915**	Victorian Infectious Diseases Reference Laboratory (VIDRL)	Victorian Infectious Diseases Reference Laboratory and Microbiological Diagnostic Unit Public Health Laboratory, Doherty Institute
**EPI_ISL_419907**	Victorian Infectious Diseases Reference Laboratory (VIDRL)	Victorian Infectious Diseases Reference Laboratory and Microbiological Diagnostic Unit Public Health Laboratory, Doherty Institute
**EPI_ISL_419902**	Victorian Infectious Diseases Reference Laboratory (VIDRL)	Victorian Infectious Diseases Reference Laboratory and Microbiological Diagnostic Unit Public Health Laboratory, Doherty Institute
**EPI_ISL_419892**	Victorian Infectious Diseases Reference Laboratory (VIDRL)	Victorian Infectious Diseases Reference Laboratory and Microbiological Diagnostic Unit Public Health Laboratory, Doherty Institute
**EPI_ISL_419887**	Victorian Infectious Diseases Reference Laboratory (VIDRL)	Victorian Infectious Diseases Reference Laboratory and Microbiological Diagnostic Unit Public Health Laboratory, Doherty Institute
**EPI_ISL_419886**	Victorian Infectious Diseases Reference Laboratory (VIDRL)	Victorian Infectious Diseases Reference Laboratory and Microbiological Diagnostic Unit Public Health Laboratory, Doherty Institute

**EPI_ISL_419885**	Victorian Infectious Diseases Reference Laboratory (VIDRL)	Victorian Infectious Diseases Reference Laboratory and Microbiological Diagnostic Unit Public Health Laboratory, Doherty Institute
**EPI_ISL_419884**	Victorian Infectious Diseases Reference Laboratory (VIDRL)	Victorian Infectious Diseases Reference Laboratory and Microbiological Diagnostic Unit Public Health Laboratory, Doherty Institute
**EPI_ISL_419877**	Victorian Infectious Diseases Reference Laboratory (VIDRL)	Victorian Infectious Diseases Reference Laboratory and Microbiological Diagnostic Unit Public Health Laboratory, Doherty Institute
**EPI_ISL_419864**	Victorian Infectious Diseases Reference Laboratory (VIDRL)	Victorian Infectious Diseases Reference Laboratory and Microbiological Diagnostic Unit Public Health Laboratory, Doherty Institute
**EPI_ISL_419859**	Victorian Infectious Diseases Reference Laboratory (VIDRL)	Victorian Infectious Diseases Reference Laboratory and Microbiological Diagnostic Unit Public Health Laboratory, Doherty Institute
**EPI_ISL_419855**	Victorian Infectious Diseases Reference Laboratory (VIDRL)	Victorian Infectious Diseases Reference Laboratory and Microbiological Diagnostic Unit Public Health Laboratory, Doherty Institute
**EPI_ISL_419845**	Victorian Infectious Diseases Reference Laboratory (VIDRL)	Victorian Infectious Diseases Reference Laboratory and Microbiological Diagnostic Unit Public Health Laboratory, Doherty Institute
**EPI_ISL_419841**	Victorian Infectious Diseases Reference Laboratory (VIDRL)	Victorian Infectious Diseases Reference Laboratory and Microbiological Diagnostic Unit Public Health Laboratory, Doherty Institute
**EPI_ISL_419837**	Victorian Infectious Diseases Reference Laboratory (VIDRL)	Victorian Infectious Diseases Reference Laboratory and Microbiological Diagnostic Unit Public Health Laboratory, Doherty Institute
**EPI_ISL_419823**	Microbiological Diagnostic Unit Public Health Laboratory	Microbiological Diagnostic Unit Public Health Laboratory
**EPI_ISL_419821**	Victorian Infectious Diseases Reference Laboratory (VIDRL)	Victorian Infectious Diseases Reference Laboratory and Microbiological Diagnostic Unit Public Health Laboratory, Doherty Institute
**EPI_ISL_419816**	Victorian Infectious Diseases Reference Laboratory (VIDRL)	Victorian Infectious Diseases Reference Laboratory and Microbiological Diagnostic Unit Public Health Laboratory, Doherty Institute
**EPI_ISL_419814**	Victorian Infectious Diseases Reference Laboratory (VIDRL)	Victorian Infectious Diseases Reference Laboratory and Microbiological Diagnostic Unit Public Health Laboratory, Doherty Institute
**EPI_ISL_419813**	Victorian Infectious Diseases Reference Laboratory (VIDRL)	Victorian Infectious Diseases Reference Laboratory and Microbiological Diagnostic Unit Public Health Laboratory, Doherty Institute
**EPI_ISL_419806**	Victorian Infectious Diseases Reference Laboratory (VIDRL)	Victorian Infectious Diseases Reference Laboratory and Microbiological Diagnostic Unit Public Health Laboratory, Doherty Institute
**EPI_ISL_419805**	Victorian Infectious Diseases Reference Laboratory (VIDRL)	Victorian Infectious Diseases Reference Laboratory and Microbiological Diagnostic Unit Public Health Laboratory, Doherty Institute
**EPI_ISL_419799**	Victorian Infectious Diseases Reference Laboratory (VIDRL)	Victorian Infectious Diseases Reference Laboratory and Microbiological Diagnostic Unit Public Health Laboratory, Doherty Institute
**EPI_ISL_419796**	Victorian Infectious Diseases Reference Laboratory (VIDRL)	Victorian Infectious Diseases Reference Laboratory and Microbiological Diagnostic Unit Public Health Laboratory, Doherty Institute
**EPI_ISL_419788**	Victorian Infectious Diseases Reference Laboratory (VIDRL)	Victorian Infectious Diseases Reference Laboratory and Microbiological Diagnostic Unit Public Health Laboratory, Doherty Institute
**EPI_ISL_419782**	Victorian Infectious Diseases Reference Laboratory (VIDRL)	Victorian Infectious Diseases Reference Laboratory and Microbiological Diagnostic Unit Public Health Laboratory, Doherty Institute
**EPI_ISL_419781**	Victorian Infectious Diseases Reference Laboratory (VIDRL)	Victorian Infectious Diseases Reference Laboratory and Microbiological Diagnostic Unit Public Health Laboratory, Doherty Institute
**EPI_ISL_419771**	Victorian Infectious Diseases Reference Laboratory (VIDRL)	Victorian Infectious Diseases Reference Laboratory and Microbiological Diagnostic Unit Public Health Laboratory, Doherty Institute

**EPI_ISL_419762**	Victorian Infectious Diseases Reference Laboratory (VIDRL)	Victorian Infectious Diseases Reference Laboratory and Microbiological Diagnostic Unit Public Health Laboratory, Doherty Institute
**EPI_ISL_419760**	Victorian Infectious Diseases Reference Laboratory (VIDRL)	Victorian Infectious Diseases Reference Laboratory and Microbiological Diagnostic Unit Public Health Laboratory, Doherty Institute
**EPI_ISL_419757**	Victorian Infectious Diseases Reference Laboratory (VIDRL)	Victorian Infectious Diseases Reference Laboratory and Microbiological Diagnostic Unit Public Health Laboratory, Doherty Institute
**EPI_ISL_419755**	Victorian Infectious Diseases Reference Laboratory (VIDRL)	Victorian Infectious Diseases Reference Laboratory and Microbiological Diagnostic Unit Public Health Laboratory, Doherty Institute
**EPI_ISL_419748**	Victorian Infectious Diseases Reference Laboratory (VIDRL)	Victorian Infectious Diseases Reference Laboratory and Microbiological Diagnostic Unit Public Health Laboratory, Doherty Institute
**EPI_ISL_419733**	Victorian Infectious Diseases Reference Laboratory (VIDRL)	Victorian Infectious Diseases Reference Laboratory and Microbiological Diagnostic Unit Public Health Laboratory, Doherty Institute
**EPI_ISL_419732**	Microbiological Diagnostic Unit Public Health Laboratory	Microbiological Diagnostic Unit Public Health Laboratory
**EPI_ISL_419727**	Microbiological Diagnostic Unit Public Health Laboratory	Microbiological Diagnostic Unit Public Health Laboratory
**EPI_ISL_419722**	Microbiological Diagnostic Unit Public Health Laboratory	Microbiological Diagnostic Unit Public Health Laboratory
**EPI_ISL_419714**	Microbiological Diagnostic Unit Public Health Laboratory	Microbiological Diagnostic Unit Public Health Laboratory
**EPI_ISL_418869**	UW Virology Lab	UW Virology Lab
**EPI_ISL_418404**	Department of Virology and Immunology, University of Helsinki and Helsinki University Hospital, Huslab Finland	Department of Virology, Faculty of Medicine, University of Helsinki, Helsinki, Finland
**EPI_ISL_418207**	Institut Pasteur Dakar	Institut Pasteur de Dakar
**EPI_ISL_418008**	H Braga	Instituto Nacional de Saude (INSA)
**EPI_ISL_418007**	H Braga	Instituto Nacional de Saude (INSA)
**EPI_ISL_417931**	UCSF Clinical Microbiology Laboratory	Chan-Zuckerberg Biohub
**EPI_ISL_417675**	The National University Hospital of Iceland	deCODE genetics
**EPI_ISL_417619**	The National University Hospital of Iceland	deCODE genetics
**EPI_ISL_417536**	deCODE genetics	deCODE genetics
**EPI_ISL_417156**	Washington State Department of Health	Seattle Flu Study
**EPI_ISL_416885**	National Public Health Laboratory	Malaysia Genome Institute
**EPI_ISL_416514**	Victorian Infectious Diseases Reference Laboratory (VIDRL)	Victorian Infectious Diseases Reference Laboratory and Microbiological Diagnostic Unit Public Health Laboratory, Doherty Institute
**EPI_ISL_416492**	University of Wisconsin-Madison AIDS Vaccine Research Laboratories	University of Wisconsin-Madison AIDS Vaccine Research Laboratories
**EPI_ISL_416467**	KU Leuven, Clinical and Epidemiological Virology	KU Leuven, Clinical and Epidemiological Virology
**EPI_ISL_416454**	UW Virology Lab	UW Virology Lab
**EPI_ISL_416413**	Victorian Infectious Diseases Reference Laboratory (VIDRL)	Victorian Infectious Diseases Reference Laboratory and Microbiological Diagnostic Unit Public Health Laboratory, Doherty Institute
**EPI_ISL_416411**	Victorian Infectious Diseases Reference Laboratory (VIDRL)	Victorian Infectious Diseases Reference Laboratory and Microbiological Diagnostic Unit Public Health Laboratory, Doherty Institute
**EPI_ISL_416409**	Shanghai Public Health Clinical Center, Shanghai Medical College, Fudan University	National Research Center for Translational Medicine (Shanghai), Ruijin Hospital affiliated to Shanghai Jiao Tong University School of Medicine & Shanghai Public Health Clinical Center
**EPI_ISL_416144**	Department of Virus and Microbiological Special diagnostics, Statens Serum Institut, Copenhagen, Denmark.	ViFU
**EPI_ISL_415625**	UW Virology Lab	UW Virology Lab
**EPI_ISL_415598**	UW Virology Lab	UW Virology Lab
**EPI_ISL_412975**	Centre for Infectious Diseases and Microbiology Laboratory Services	NSW Health Pathology - Institute of Clinical Pathology and Medical Research
